# Proteomic profiling of *Rhizobium tropici* PRF 81: identification of conserved and specific responses to heat stress

**DOI:** 10.1186/1471-2180-12-84

**Published:** 2012-05-30

**Authors:** Douglas Fabiano Gomes, Jesiane Stefânia da Silva Batista, Aline Luiza Schiavon, Diva Souza Andrade, Mariangela Hungria

**Affiliations:** 1 Embrapa Soja, C.P. 231, 86001-970, Londrina, PR, Brazil; 2 Departamento de Biotecnologia, Universidade Estadual de Londrina, C.P. 6001, 86055-990, Londrina, Brazil; 3 Centro Universitário Filadélfia (UNIFIL), C.P. 1626, 86020-000, Londrina, Brazil; 4 Instituto Agronômico do Paraná (IAPAR), C.P. 481, 86001-970, Londrina, Brazil

## Abstract

**Background:**

*Rhizobium tropici* strain PRF 81 (= SEMIA 4080) has been used in commercial inoculants for application to common-bean crops in Brazil since 1998, due to its high efficiency in fixing nitrogen, competitiveness against indigenous rhizobial populations and capacity to adapt to stressful tropical conditions, representing a key alternative to application of N-fertilizers. The objective of our study was to obtain an overview of adaptive responses to heat stress of strain PRF 81, by analyzing differentially expressed proteins when the bacterium is grown at 28°C and 35°C.

**Results:**

Two-dimensional gel electrophoresis (2DE) revealed up-regulation of fifty-nine spots that were identified by MALDI-TOF/TOF-TOF. Differentially expressed proteins were associated with the functional COG categories of metabolism, cellular processes and signaling, information storage and processing. Among the up-regulated proteins, we found some related to conserved heat responses, such as molecular chaperones DnaK and GroEL, and other related proteins, such as translation factors EF-Tu, EF-G, EF-Ts and IF2. Interestingly, several oxidative stress-responsive proteins were also up-regulated, and these results reveal the diversity of adaptation mechanisms presented by this thermotolerant strain, suggesting a cross-talk between heat and oxidative stresses.

**Conclusions:**

Our data provide valuable protein-expression information relevant to the ongoing genome sequencing of strain PRF 81, and contributes to our still-poor knowledge of the molecular determinants of the thermotolerance exhibited by *R. tropici* species.

## Background

In most agricultural soils, nitrogen (N) is the main limiting nutrient and, accordingly, it is often supplied to crops as chemical fertilizers. Significant losses of N-fertilizers occur either by leaching—resulting in eutrophication of rivers, lakes, aquifers— or by denitrification, contributing to global warming [1]. However, estimates indicate that up to 60% of the N needs of legume crops may be obtained from the biological nitrogen fixation (BNF) process [2,3], with significant economic benefits to farmers while mitigating environmental impacts.

Common bean (*Phaseolus vulgaris* L.) is the most important food legume in South and Central America and in East Africa. It can establish symbiotic relationships with a variety of described and still-to-be-described rhizobial species [4]. An important limitation to the BNF process involving common bean is the high genetic instability of the symbiotic plasmid of the rhizobial strains, as reported for *Rhizobium phaseoli* and *Rhizobium etli*. This instability has been attributed to genomic rearrangements, plasmid deletions and mutations, which are intensified under stressful conditions [5,6]. Abiotic stresses such as high soil temperatures, in addition to water deficit, salinity and soil acidity comprise the main factors causing genetic instability [7,8].

Among common-bean rhizobia, *Rhizobium tropici* is recognized for its tolerance of environmental stresses, including high temperatures [7-9]. Within this species, strain PRF 81 (= SEMIA 4080) is known for the high capacity in fixing N_2_, competitiveness against other rhizobia, and tolerance of environmental stresses; it has been used in commercial inoculants in Brazil since 1998 [10,11]. More information about the strain, including genetic characterization, is given elsewhere [10,12,13]. The strain is deposited at the *“*Diazotrophic and Plant Growth Promoting Bacteria Culture Collection*”* at Embrapa Soja ( http://www.bmrc.lncc.br).

Mechanisms of response to stresses are usually highly conserved among bacterial species, and designed for rapid adaptation to environmental and metabolic changes. These conserved responses comprise the expression of molecular chaperones, such as DnaK (and its assistants DnaJ and GrpE), GroEL (and its assistant GroES), and also of small heat-shock proteins [14]. All are polypeptide-binding proteins implicated in protein folding, protein targeting to membranes, renaturation, and in the control of protein-protein interactions. In addition to conserved responses, some bacterial species also possess specific metabolic adaptations to stressful conditions.

Recently, a draft genome of *R. tropici* strain PRF 81 revealed several probable genes that may be related to its outstanding symbiotic and saprophytic abilities and also its adaptability to environmental stresses [12]; elucidation of the whole genome of the strain is now in progress ( http://www.bnf.lncc.br). However, elucidating biological implications of a given genome requires understanding of gene expression; therefore, proteomic studies, complementary to the structural genome, are critical.

Despite the ecological, evolutionary and economic importance of *R. tropici*, proteomic information about the species is scarce. In addition, the intriguing tolerance to high temperature of *R. tropici* strains is far from being understood. In this context, our objective with this study was to report a proteomic study of *R. tropici* strain PRF 81, focusing on the determination of adaptive responses to heat stress.

## Methods

### Bacterial growth conditions

*R. tropici* strain PRF 81 was pre-cultured in 10-mL aliquots of tryptone-yeast extract medium (TY), at 80 rpm and 28°C, in the dark. The pre-cultures were then transferred to Erlenmeyer flasks containing 200 mL of TY medium and bacteria were grown under two treatment conditions: control (28°C) and with heat stress (35°C). Cells were incubated until the exponential phase of growth was reached (optical density of 0.6 at 600 nm), what took approximately 18 h, with low agitation (80 rpm) to minimize the production of extra-cellular polysaccharides, which can interfere in 2-D gel electrophoresis.

### Total protein extraction

Cultures were centrifuged at 5,000 x g, at 4°C and cells were carefully washed with a solution containing 3 mM KCl; 1.5 mM KH_2_PO_4_; 68 mM NaCl; and 9 mM NaH_2_PO_4_. Washed cells were resuspended in 600 μL of a buffer containing 10 mM Tris–HCl pH 8.0; 1.5 mM MgCl_2_; 10 mM KCl; 0.5 mM DTT; and 0.5 mM PMSF. Aliquots of 150 μL were stored in ultrafreezer (–80°C) until the analyses.

For whole-cell protein extraction, aliquots were resuspended in lysis buffer containing 9.5 M urea; 2% CHAPS; 0.8% v/v Pharmalyte 4–7; and 1% DTT, and submitted to forty cycles of freezing in liquid N_2_ and thawing at 37°C, as described by Lery *et al.*[15]. The lysates were separated from particulate material by centrifugation at 14.000 x g for 90 min, at 4°C.

An additional step of concentration with phenol was done, increasing significantly the quality and reproducibility of the 2-D gels (data not shown). Aliquots (500 μL) of the lysates were homogenized with a solution containing 0.8 mL of Tris-buffered phenol pH 8.0, and 0.8 mL of SDS buffer (0.1 M Tris–HCl pH 8.0; 2% SDS; 5% β-mercaptoethanol; 30% sucrose; 1 mM phenylmethylsulfonyl fluoride, PMSF). The samples were homogenized for 5 min and centrifuged at 16,000 x g for 15 min at 4°C, and the top phenol layer (500 μL) was transferred to a new tube. Proteins were precipitated for 1 h at –20°C with three volumes of pre-cooled 0.1 M ammonium acetate in absolute methanol and then centrifuged (16,000 x g for 15 min at 4°C). The pellet was washed once with pre-cooled methanol and once with pre-cooled 80% v/v acetone, followed by drying.

The pellet was resuspended with the lysis buffer and concentration was determined by Bradford’s method [16].

### 2-D electrophoresis and visualization

For IEF, lysates were dissolved with DeStreak buffer (GE Healthcare) to a final concentration of 300 μg of protein and 2% v/v IPGphor in 250 μL of solution. IPG-strips (pH 4–7, 13 cm, GE Healthcare) were rehydrated with the protein solution and covered with cover fluid (GE Healthcare). Loaded strips were submitted to focalization in an Ettan IPGphor IEF system (GE Healthcare) for 1 h at 200 V, 1 h at 500 V, a gradient step to 1,000 V for 1 h, a gradient step to 8,000 V for 2 h 30 min, and fixed at 8,000 V for 1 h 30 min. The final Vh was fixed at 24,800. After focusing, strips were equilibrated first for 20 min in 5 mL of TE buffer (50 mM Tris–HCl pH 8.8; 6 M urea; 30% v/v glycerol; 2% w/v SDS; and 0.2% v/v of a 1% solution of bromophenol blue) supplemented with 50 mg DTT and then in TE buffer with 175 mg iodoacetamine, also for 20 min.

2-D electrophoresis was performed on a 12% polyacrylamide gel (18 × 16 cm) in a Ruby SE 600 vertical electrophoresis system (GE Healthcare). The run was carried out for 30 min at 15 mA/gel and 240 min at 30 mA/gel, using the Low Molecular Weight Calibration Kit for SDS Electrophoresis (Amersham Biosciences) to provide standards. For each strain, the extraction procedure and gel electrophoresis were run in triplicates. Gels were fixed overnight with an ethanol-acetic acid solution before being stained with Coomassie Blue PhastGel^TM^ R-350 (GE Healthcare) and scanned (ImageScanner LabScan v5.0).

### Gel image analysis and spot selection

Spots were strictly identified in the high-resolution digitalized gel images and analyzed by Image Master 2D Platinum v 5.0 software (GE Healthcare). After background subtraction, ratios of mean normalized spot volumes were calculated and values of related spots were compared between both conditions. All selected spots exhibiting a higher volume in the heat stress condition were statistically evaluated (*p* ≤ 0.05) upon Student’s *t*-test, using XLSTAT (Addinsoft, France, add-in to Microsoft Excel).

### Sample preparation and MALDI-TOF mass spectrometry

Protein spots showing significant changes in mean normalized volume were excised and processed as described by Chaves *et al.*[17]. Digestion was achieved with trypsin (Gold Mass Spectrometry Grade, Promega, Madison, WI), at 37°C, overnight.

Tryptic peptides (1 μL) were mixed with saturated solution of α-cyano- 4-hydroxy-cinnamic acid (HCCA) in 50% acetonitrile, 0.1% trifluoroacetic acid (TFA). The mixture was spotted onto a MALDI (matrix assisted laser desorption ionization) sample plate and allowed to crystallize at room temperature. The same procedure was used for the standard peptide calibration mix (Bruker Daltonics). For mass spectra acquisition, a MALDI-TOF-MS (MALDI-time-of-flight in tandem) Autoflex Spectrometer (Bruker Daltonics) was operated in the reflector for MALDI-TOF peptide mass fingerprint (PMF) and in the “LIFT” mode for MALDI-TOF/TOF in the fully manual mode, using FlexControl 3.0 software.

### *In silico* protein identification

PMFs and MS/MS ion spectra generated were searched against the public database NCBInr (National Center for Biotechnology Information non-redundant), using Mascot software v. 2.3 (Matrix Science). For protein searches, performed in the Proteobacteria taxonomic group, monoisotopic masses were used, considering a peptide tolerance of 150 ppm and allowance of one missed cleavage. When MS/MS was carried out, a tolerance of 0.3 Da was acceptable. Carbamidomethylation of cysteine and oxidation of methionine were considered fixed and variable modifications, respectively.

Identifications were validated only when the Mowse (molecular weight search) score was significant, above the recommended cutoff of 52 for PMFs. Searches on the Decoy database were done in the automated mode in the Mascot software, using a random database (NCBInr/Proteobacteria) strategy. Both decoy score and false discovery rates were considered for the validation of the searches of MS and MS/MS data and to measure the quality of the matches (*p ≤* 0.05); using this approach false discovery rates were always less than 1%. The spectrometry datasets are available at PRIDE ( http://ebi.ac.uk/pride/) with the experiment accession number 14817.

### Protein characterization

A set of bioinformatics tools was used for improved characterization of identified proteins. The proteins were fitted into COG (Clusters of Orthologous Groups) categories according to their functional inference, using the COGnitor program ( http://www.ncbi.nih.gov/COG) [18]. Software packages PSORT-B [19] and PSLpred [20] were used for prediction of subcellular localization.

## Results and discussion

### 2-D electrophoresis and differential spots selection

Several compounds, such as salts, nucleic acids and polysaccharides, may interfere with electrophoretic separation, resulting in streaky 2-D patterns, and thus should be removed. *R. tropici* PRF 81 produces high amounts of exopolysaccharides (EPS) *in vitro* and interference with electrophoretic resolution was overcome with a final wash step of the whole protein extract with phenol. In addition, to improve separation resolution, we employed IPG strips with a pH range of 4.0 to 7.0 in the first-dimension electrophoresis, to achieve better protein resolution than with broader-range (pH 3.0 to 10.0) strips (data not shown), in which the proteins remained concentrated in the central part of the gel (pH 5.0 to 7.0).

**Figure 1:**
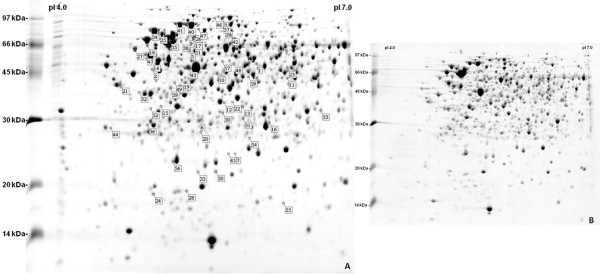
**Whole cell 2DE protein gel profiles of*Rhizobium tropici*PRF 81.** For analysis of heat stress response on protein expression, 2DE gel profiles of *R. tropici* grown at 35°C (A) and 28°C (B) were obtained. More information about differential expressed proteins assigned is available in Table 1 and Additional file 1: Table S1.

Using the computer-assisted gel-image analysis software, the majority of the molecular masses associated with the spots ranged between 14 and 97 kDa (Figure 1). The volume of each spot was normalized as a percent of the total volume of all detected spots in the gel. This procedure was followed for all gels and the values generated for each spot were compared between the control (28°C) and the experimental (35°C) treatment, and only well-defined spots present in the three replicates and showing statistically significant differences (*p* ≤ 0.05) were selected. A significant difference in several relative spot volumes indicated that the elevated temperature led to changes in the proteome of *R. tropici* PRF 81.

### General proteome response to heat stress

Maximum soil temperatures in tropical soils can often exceed 40°C. Optimal temperature of growth of *R. tropici* species is around 28°C, and although there are reports of tolerance of PRF 81 to 40°C [9,10], our preliminary tests have shown that 35°C was the highest temperature that did not affect substantially growth; under higher temperatures, the slower growth rate had critical effects on the proteomic profile (data not shown). Joszefczuk *et al.*[21] also reported, in a heat stress response experiment with *Escherichia coli,* that one of the most striking features was the strong influence of high temperatures on the bacterium growth. In addition, contrasting with the majority of the studies about heat stress only with a short period of growth at high temperatures, our study considered a heat stress for the whole period of PRF 81 growth.

In comparison to other common-bean rhizobial species, *R. tropici* is known for its genetic stability and adaptation to stressful conditions [8,9], and, although PRF 81 is an outstanding strain in terms of these properties [10,11,13], little is known of the molecular determinants of its heat tolerance. In order to obtain an overview of the heat responses, we analyzed the cytoplasmic and periplasmic contents and identified the whole-cell protein expression changes when the cells were grown at 35°C. Fifty-nine significantly induced proteins were identified by mass spectrometry, and twenty-six of them were detected exclusively under heat stress conditions. All identified proteins were distributed across fifteen COG functional categories; six fit into the category of general prediction (R), one was classified in the category of unknown function (S) and only one was assigned as “not in COG” (Table 1).

**Table 1:** **Identified proteins of*Rhizobium tropici*PRF 81 whole cell extracts up-regulated after growth at high temperature (35°C)**

**Spot ID**	**NCBI ID**	**Gene**	**Protein description**	**Organism (best match)**	**T/E**^**1**^** pI**	**T/E**^**1**^**mass (Da)**	**Fold change ratio**^**2**^	***p-value***	**Cellular location**
Metabolism									
C - Energy production and conversion									
1	gi|46909738	*icd*	Isocitrate dehydrogenase	*Rhizobium leguminosarum*	5.9/5.96	45320/49000	↑1.00	-	Cytoplasmic
2	gi|222087461	*sucC*	Succinyl-coa synthetase beta subunit protein	*Agrobacterium radiobacter*	4.98/4.96	42028/46000	3.27 ± 0.12	0.001	Cytoplasmic
3	gi|86359524	*acnA*	Aconitate hydratase	*Rhizobium etli*	5.48/5.69	97180/98000	1.65 ± 0.06	0.001	Cytoplasmic
4	gi|116254139	*atpD*	F0F1 ATP synthase subunit beta	*Rhizobium leguminosarum*	5.03/4.88	50885/56000	2.68 ± 0.03	0.001	Cytoplasmic
E- Amino acid transport and metabolism									
5	gi|1245379	*glnA*	Glutamine synthetase I	*Sinorhizobium meliloti*	5.2/5.33	52287/61000	2.92 ± 0.08	0.001	Cytoplasmic
6	gi|15887731	*argB*	Acetylglutamate kinase	*Agrobacterium tumefaciens*	5.16/5.41	31083/30000	2.19 ± 0.09	0.001	Cytoplasmic
7	gi|89258357		Putative periplasmic substrate binding protein	*Ochrobactrum anthropi*	5.84/5.78	28188/24000	↑1.00	-	Periplasmic
8	gi|222109054	*nocP*	Opine permease ATP-binding protein	*Agrobacterium radiobacter*	6.98/5.22	28288/20000	↑1.00	-	Inner Membrane
9	gi|222087066	*pepF*	Oligoendopeptidase F protein	*Agrobacterium radiobacter*	5.32/5.33	68989/76000	↑1.00	-	Cytoplasmic
10	gi|222087908	*asd*	Aspartate-B-semialdehyde dehydrogenase protein	*Agrobacterium radiobacter*	5.46/5.59	37925/45000	1.38 ± 0.043	0.001	Cytoplasmic
11	gi|222084786	*argD*	Diaminobutyrate--pyruvate aminotransferase protein	*Agrobacterium radiobacter*	5.63/6.35	42909/43000	↑1.00	-	Cytoplasmic
12	gi|114765810	*ilvE*	Branched-chain amino acid aminotransferase	*Pelagibaca bermudensis*	5.31/5.68	32142/35000	↑1.00	-	Cytoplasmic
F- Nucleotide transport and metabolism									
13	gi|86146888	*pyrH*	Uridylate Kinase	*Vibrio* sp.	5.08/5.82	26284/33000	1.38 ± 0.13	0.008	Cytoplasmic
G - Carbohydrate transport and metabolism									
14	gi|222085874	*eno*	Phosphopyruvate hydratase	*Agrobacterium radiobacter*	4.84/4.95	45120/53000	2.88 ± 0.37	0.005	Cytoplasmic
15	gi|282887091		Alpha amylase catalytic region	*Burkholderia sp.*	6.26/5.03	64245/34000	↑1.00	0.001	Cytoplasmic
16	gi|241206422		Transaldolase	*Rhizobium leguminosarum*	5.32/6.12	35091/29000	↑1.00	-	Cytoplasmic
17	gi|11493200	*pgm*	Phosphoglucomutase	*Rhizobium tropici*	5.16/5.38	58641/72000	↑1.00	-	Cytoplasmic
18	gi|222084905	*aglA*	Alpha-glucosidase protein	*Agrobacterium radiobacter*	4.84/4.86	62592/65000	↑1.00	-	Cytoplasmic
H - Coenzyme transport and metabolism									
19	gi|222086485		ABC transporter	*Agrobacterium radiobacter*	5.23/5.21	38975/42000	1.70 ± 0.09	0.001	Periplasmic
20	gi|296105270		Biotin protein ligase	*Enterobacter cloacae*	5.23/5.42	35255/28000	3.98 ± 0.24	0.001	Cytoplasmic
I - Lipid transport and metabolism									
21	gi|299768808		Acyl-coa dehydrogenase	*Agrobacterium tumefaciens*	5.37/4.66	65994/40000	↑1.00	-	Cytoplasmic
22	gi|282888281		3-Oxoacyl-(acyl-carrier-protein (ACP)) synthase III domain protein	*Burkholderia* sp.	6.27/5.74	38552/35000	↑1.00	-	Cytoplasmic
23	gi|159186213	*pcaF*	Beta-ketoadipyl coa thiolase	*Agrobacterium tumefaciens*	5.51/6.37	41850/46000	2.95 ± 0.07	0.001	Cytoplasmic
*P - Inorganic ion transport and metabolism*									
24	gi|222087891	*bfr*	Bacterioferritin	*Agrobacterium radiobacter*	4.81/4.94	16860/19000	2.27 ± 0.07	0.001	Cytoplasmic
25	gi|87199081		Tonb-dependent receptor	*Novosphingobium aromaticivorans*	5.82/5.01	87810/75000	↑1.00	-	Extra Cellular
Cellular processes and signaling									
D - Cell cycle control, cell division, chromosome partitioning									
26	gi|222086436	*ftsZ2*	Cell division protein	*Agrobacterium radiobacter*	5.21/5.39	63014/81000	2.42 ± 0.26	0.003	Cytoplasmic
27	gi|50121473	*kicB*	Condesin subunit F	*Pectobacterium atrosepticum*	4.7/4.78	50717/57000	↑1.00	-	Cytoplasmic
T - Signal transduction mechanisms									
28	gi|117926246		Protein tyrosine phosphatase	*Magnetococcus sp*	6.29/5.28	18731/19000	↑1.00	-	Cytoplasmic
29	gi|222087232	*prkA*	Serine protein kinase protein	*Agrobacterium radiobacter*	5.42/5.69	74417/84000	2.41 ± 0.19	0.001	Cytoplasmic
30	gi|116252038	*ntrX*	Putative two component response regulator Nitrogen assimilation regulatory protein	*Rhizobium leguminosarum*	9.15/5.66	30427/34000	↑1.00	-	Cytoplasmic
31	gi|159184131	*chvI*	Two component response regulator	*Agrobacterium tumefaciens*	5.56/5.85	27253/30000	1.35 ± 0.10	0.003	Cytoplasmic
O - Posttranslational modification, protein turnover, chaperones									
32	gi|222087564	*trxA*	Thioredoxin	*Agrobacterium radiobacter*	4.83/4.85	34469/39000	↑1.00	-	Cytoplasmic
33	gi|118590060	*bcp*	Bacterioferritin comigratory protein	*Stappia aggregata*	5.63/5.37	16749/22000	3.40 ± 0.26	0.001	Cytoplasmic
34	gi|58826564	*dnaK*	Dnak	*Rhizobium tropici*	4.91/5.37	68393/74000	↑1.00	-	Cytoplasmic
35	gi|222085003	*groEL*	Chaperonin GroEL	*Agrobacterium radiobacter*	5.03/5.11	57836/69000	1.36 ± 0.19	0.012	Cytoplasmic
M - Cell wall/membrane/envelope biogenesis									
36	gi|86359655		Putative metalloendopeptidase protein	*Rhizobium etli*	5.36/4.89	49514/29000	1.31 ± 0.22	0.02	Periplasmic
37	gi|222085864	*omp1*	Outer membrane lipoprotein	*Agrobacterium radiobacter*	5.26/5.66	84589/90000	↑1.00	-	Extra Cellular
N - Cell motility									
38	gi|18033179	*virD4*	VirD4	*Agrobacterium tumefaciens*	6.82/5.24	73380/69000	1.21 ± 0.16	0.024	Cytoplasmic
Information storage and processing									
J - Translation, ribosomal structure and biogenesis									
39	gi|222085858	*tsf*	Translation elongation factor Ts	*Agrobacterium radiobacter*	5.15/5.14	32268/40000	1.86 ± 0.02	0.001	Cytoplasmic
40	gi|227821753	*fusA*	Elongation factor G	*Rhizobium* sp.	5.17/5.3	77966/89000	1.98 ± 0.13	0.001	Cytoplasmic
41	gi|86355771	*pnp*	Polynucleotide phosphorylase/polyadenylase	*Rhizobium etli*	5.2/5.19	77491/89000	2.23 ± 0.09	0.001	Cytoplasmic
42	gi|294624706	*infB*	Translation initiation factor IF-2	*Xanthomonas fuscans*	5.89/5.79	83626/75000	1.29 ± 0.09	0.003	Cytoplasmic
43	gi|218672404	*tufB1*	Elongation factor EF-Tu protein	*Rhizobium etli*	4.87/5.31	31884/48000	3.40 ± 0.31	0.0024	Cytoplasmic
K – Transcription									
44	gi|89056301		LysR family transcriptional regulator	*Jannaschia* sp.	5.574.48	32077/28000	↑1.00	-	Cytoplasmic
45	gi|159184760		AraC family transcriptional regulator	*Agrobacterium tumefaciens*	7.11/5.74	27498/25000	↑1.00	-	Cytoplasmic
46	gi|222081230		Transcriptional regulator protein	*Agrobacterium radiobacter*	6.38/5.6	98220/98000	4.71 ± 0.09	0.001	Cytoplasmic
47	gi|190895600		Probable transcriptional	*Rhizobium etli*	6.91/5.42	42937/85000	↑1.00	-	Cytoplasmic
48	gi|222106418		Transcriptional regulator GntR family	*Agrobacterium vitis*	5.82/5.78	26366/49000	↑1.00	-	Cytoplasmic
49	gi|222106466		Transcriptional regulator ROK family	*Agrobacterium vitis*	7.03/5.14	41156/42000	↑1.00	-	Cytoplasmic
50	gi|222082875		Transcriptional regulator, MarR family	*Agrobacterium radiobacter*	5.46/5.57	18141/20000	↑1.00	-	Cytoplasmic
*L - Replication, recombination and repair*									
51	gi|222084927		ATP-dependent RNA helicase protein	*Agrobacterium radiobacter*	9.17/5.36	69955/67000	2.29 ± 0.14	0.001	Cytoplasmic
Poorly characterized									
R - General function prediction only									
52	gi|222086102	*sufC*	FeS assembly ATPase SufC	*Agrobacterium radiobacter*	5.08/4.95	27375/32000	↑1.00	-	Inner Membrane
53	gi|222082138	*cpo*	Chloride peroxidase protein	*Agrobacterium radiobacter*	7.88/6.37	34965/32000	1.59 ± 0.02	0.001	Periplasmic
54	gi|186472508	*wrbA*	Flavoprotein WrbA	*Burkholderia phymatum*	6.19/5.91	20930/26000	2.58 ± 0.14	0.001	Cytoplasmic
55	gi|170699364		NADPH-dependent FMN reductase	*Burkholderia ambifaria*	6.71/6.31	8539/17000	2.03 ± 0.19	0.002	Periplasmic
56	gi|194431754	*dkgA*	2,5-diketo-D-gluconic acid reductase A	*Shigella dysenteriae*	6.22/5.15	19399/23000	1.34 ± 0.21	0.002	Cytoplasmic
57	gi|222085370		Ferredoxin reductase protein	*Agrobacterium radiobacter*	5.88/5.65	43777/53000	1.48 ± 0.12	0.003	Cytoplasmic
S - Function Unknown									
58	gi|222149801		Hypothetical protein Avi_3814	*Agrobacterium vitis*	5.03/5.01	24632/29000	1.42 ± 0.34	0.033	Periplasmic
NO related COG									
59	gi|209547526		Hypothetical protein Rleg2_5527	*Rhizobium leguminosarum*	6.02/5.89	33584/44000	1.57 ± 0.13	0.002	Cytoplasmic

^1^Theoretical/Experimental values. Da: Daltons.

^2^↑1.00 in the fold change ratio means that the protein was only identified in the experimental condition (35°C).

Matched peptides masses and MS/MS combined results are available in PRIDE ( http://ebi.ac.uk/pride/) under the experiment accession number 14817.

Among the differentially expressed proteins, twenty-five were related to metabolic functions, the majority of them associated with amino acid transport and metabolism (group E) (Table 1), corroborating the proteomic reference map of *Bradyrhizobium japonicum* strain CPAC 15, a microsymbiont of soybean [22], and indicating high metabolic activity even under stressful conditions.

Also within this category, it is worth mentioning that NocP, an opine permease ATP-binding protein, was differentially expressed under high temperature. Opine is a compound released by crown-gall tumors produced by *Agrobacterium* (=*Rhizobium*) [23], and genes related to its metabolism were detected in the draft genome of PRF 81 and now confirmed at the translational level in our study. Putative genes related to rhizopine metabolism (an opine-like compound) were reported in *R. tropici* for the first time by our research group [12]. The ability to catabolize rhizopine appears to enhance the rate at which a strain is able to form nodules when it is in competition with a strain that is unable to catabolize a rhizopine. The mechanism responsible for this enhanced symbiotic ability is still unclear [24]. Moreover, we were unable to establish an exact association between stressful conditions and opine-like compounds metabolism, but our results presented an indicative of this correlation.

In relation to cellular processes and signaling, thirteen proteins were identified in categories D, T, O, M and N (Table 1). Two of these proteins are known to be correlated with heat tolerance, DnaK and GroEL molecular chaperones [12,25]. Two proteins also found in this group were thioredoxin TrxA and bacterioferritin comigratory proteins (Bcp), which have been characterized as oxidative-stress responsive.

Still considering the COG classification, thirteen induced proteins comprised a set related to information storage and processing (Table 1), including transcription regulators and translation factors. The translation factors can act as chaperones in response to heat stress, and more details of this function are discussed below.

Interesting was also the differential expression of VirD4, a TraG-like protein that plays an important role in conjugative transfer showing high similarity to *Agrobacterium*, and also reported in the draft genome of strain PRF 81 [13]. The transcription of the *vir* regulon in *Agrobacterium tumefaciens* is induced by specific plant-phenolic compounds, but also by several other abiotic stimuli, such as low pH and temperatures below 30°C [26]. VirD4 acts in the translocation of effectors proteins and has been associated with different plant-bacterium interactions, both pathogenic and symbiotic. Also, VirD4 acts in couple DNA processing and transference by conjugation mechanism. Therefore, this protein has a broader role than the action in type IV secretion system. An association between heat stress and type IV secretion system components was described by Zahri *et al.*[27], since the expression of type IV secretion system in a modified *E. coli* induced heat shock genes.

### Differential expression of the two-component response regulators (NtrX and ChvI)

Two-component systems are composed by a sensor kinase protein that transmits the environmental stimulus to a response regulator protein via phosphorylation. The phosphorylated regulator modulates the expression of the target genes required for the appropriate changes, mediating rapid metabolic responses for adaptation to new conditions [28]. Interestingly, these two up-regulated proteins in our study (NtrX and ChvI) are the response-regulator components.

NtrX has also been found to be expressed in *Gluconacetobacter diazotrophicus*[29], *Sinorhizobium* (=*Ensifer*) *meliloti*[30], and *Mesorhizobium loti*[31]. This protein is recognized to be involved in N metabolism and nitrogen fixation, probably acting as a transcriptional activator of genes related to nitrate metabolism [32,33].

The second two-component system, ChvI, characterized in several bacteria such as *S. meliloti*[34] and *A. tumefaciens*[35], acts in translation regulation of enzymes related to the biosynthesis of the succinoglycan exopolysaccharide (EPSI). In addition to this role, this two-component system signaling is critical for the viability of free-living *S. meliloti* strains [36], by acting in biofilm formation, motility, nutrient utilization and cell protection [37-39]. It has been reported that the succinoglycan may form a diffusion barrier, protecting against oxidative stress [40], suggesting that, in *R. tropici* PRF 81, in addition to participating in symbiosis signaling, the succinoglycan EPSI plays an important role in heat-stress protection.

### Induced molecular chaperones DnaK and GroEL

Temperature is especially harmful to cells because it can damage the structure of macromolecules. Many of the molecular chaperons—such as DnaK and GroEL—are highly conserved in evolution [41], preventing and repairing harmful effects. As reported in other proteomic studies [42-44], DnaK and GroEL were significantly induced in PRF 81 at high temperature.

DnaK is classified according to its molecular weight in the Hsp70 chaperone group, the most versatile chaperone system. In addition to a main role in *de novo* folding, DnaK has various other functions, including protein transport [45], and in the increased stability of RNA polymerase σ^32^ factor (RpoH), an important component of the heat-shock response in several organisms [46-49].

At optimal temperature, σ^32^ factor is rapidly degraded, but if temperature is raised, σ^32^ stability increases due to its interaction with DnaK chaperone [50]. Therefore, in response to a sudden increase in temperature, the levels of σ^32^ in the cell rise, leading to the regulation of transcription of genes encoding other heat-shock proteins, which also contribute to heat tolerance [51].

As described for *E. coli*[52]*, Bacillus cereus*[53] and *Acinetobacter baumannii*[54], in *R. tropici* PRF 81 the molecular chaperone GroEL was up-regulated under high temperature. The differential expression of GroEL is critical to thermotolerance, since the chaperone can routinely rescue more than 80% of a denatured protein population [55]. Essentially, GroEL modulates its affinity for folding intermediates through the binding and hydrolysis of ATP, and the highly coordinated binding and releasing of substrate proteins may lead to recovery of the functional state of the proteins [56].

### Induction of chaperone-like proteins: Translation factors

Besides the main function of ensuring gene expression accuracy by transporting the correct codons in the translation process, elongation and initiation factors can also act as chaperones in response to heat stress [57,58]. In our study, three elongation factors (EF-Tu, Ef-G and Ef-Ts) and one initiation factor (IF-2) were up-regulated when *R. tropici* PRF 81 was grown at 35°C (Table 1), indicating the probable involvement of these factors in protein folding and protection, contributing to the thermotolerance of PRF 81.

EF-Tu is highly homologous to cellular GTP-proteins, occupying a key position in translation [59]. EF-Tu interacts with GTP, aminoacyl-tRNA, ribosomes, and a second factor, EF-Ts, which mediates GDP/GTP exchange on EF-Tu. In addition, Hendrick, and Hartl [60] observed that EF-Tu protein may also act as a molecular chaperone, protecting proteins against thermal damage. Studying heat responses, Jacobson and Rosenbuch [61] reported that large quantities of EF-Tu molecules in cells might constitute a reservoir of chaperone-like molecules that prevent the aggregation of non-native proteins until permissive renaturation conditions are restored. The shift of the activities of transport of aminoacyl-tRNA to the aminoacyl ribosome site and as chaperone of EF-Tu is dependent on the binding of this factor with GTP or GDP.

Considering the efficiency of chaperone activity, [57] showed that the elongation factor EF-Tu when bonded with GDP had greater capacity of stimulating renaturation of enzymes than when interacting with GTP. In contrast, Kudlicki and collaborators [62] found that EF-Tu bonded with GDP is less active than when it is bonded with GTP in catalyzing protein renaturation. Still, in that study, the authors reported that the EF-Ts elongation factor plays a similar role as GTP, suggesting that in the presence of these cofactors—EF-Ts or GTP—EF-Tu can perform several rounds of protein renaturation. These divergent studies indicate that the EF-Tu chaperonin activity is dependent on the specific protein in which the protection will be promoted. Interestingly, in our study, both elongation factors—EF-Tu and EF-Ts—were up-regulated under heat stress.

Both the elongation factor EF-G and the initiation factor IF2 were also found to act as chaperone proteins [58]. These factors are involved in the translocation of ribosomes on mRNA and in the binding of initiator tRNA to the 30 S ribosomal subunit, respectively [63]. EF-G bound to GDP, instead of to GTP, seems to be more active in the formation of stable complexes with unfolded proteins, assisting in protein folding and renaturation [52]. Finally, the chaperone properties of EF-Tu, EF-G, and IF2 suggest that translation factors are ancestral protein-folding factors that appeared before chaperones and protein-disulfide isomerases [58].

### Cross-talk between heat and oxidative stress

Reactive oxygen species (ROS) are by-products of normal metabolic processes, but at high levels may be lethal for cells. However, in both symbiotic and pathogenic relations, transient production of ROS, detected in the early events of plant-microorganism interactions, may be considered as specific signals during the interaction process [64]. Previous studies have reported the accumulation of ROS in early stages of *Rhizobium*/legumes symbiosis establishment [65-67]. Therefore, the ability of the bacteria to tolerate and overcome the changes in the environment induced by the plant host seems to be important for the establishment of a successful symbiotic interaction [68].

To detoxify ROS, symbiotic bacteria display a multiple antioxidant defense that is required for both the development and the functioning of the symbiosis [69]. Fernando *et al.*[70] showed participation of thioredoxin (TrxA) in the reactivation of proteins damaged by oxidative stress, or by other conditions that cause the generation of ROS. These compounds cause covalent modifications in proteins, for example the oxidation of free sulfydryl groups (-SH), forming disulfide bonds (S-S). In this case, thioredoxin transfers reducing power to damaged proteins, restoring their reduced state [71]. Finally, thioredoxin was synthesized under high-temperature conditions, confirming its induction as a general response to stress [72]; it is also induced in the early stages of symbiotic interaction in *B. japonicum*[73] and in the plant interaction with *G. diazotrophicus*[74].

Both bacterioferritin (Bfr), a protein related to inorganic ion transport, and bacterioferritin comigratory protein (Bcp), a peroxiredoxin protein, were up-regulated in our study. These proteins have been related to oxidative stress responses, similarly to thioredoxin. The former (Bfr) acts indirectly in defense mechanisms against oxidative damage effects inside the cell, since it transports inorganic ions, for example Fe^2+^, resulting in the decomposition of the peroxides over-produced during the oxidative stress [70]. The latter (Bcp) has a protective role in the defensive response to oxidative stress, possibly via up-regulation of total and reduced glutathione levels [75].

In *Salmonella typhimurium*, the oxidative stress caused by hydrogen peroxide treatment led to the induction of heat shock proteins such as DnaK, while the heat stress induced proteins related with cell protection against the oxidative stress [76]. Interestingly, when Lenco *et al.*[77] studied oxidative stress responses from a proteomic perspective, they observed the induction of several heat-responsive proteins, such as GroEL and GroES, as a reflection of regulation of heat-shock protein biosynthesis during bacterial oxidative stress.

We found up-regulation of several proteins responsive to oxidative stress, such as isocitrate dehydrogenase, which plays a key role in NADPH recycling under oxidative stress [78-80]**]**, also the flavoprotein WrbA, a quinone oxidoreductase with redox activity [80,81], among others. These results, added to others reporting the expression of heat responsive proteins during the oxidative stress, suggest a cross-talk between heat stress and oxidative stress responses.

## Conclusions

Although most of the proteins involved in responses to heat are highly conserved, the regulatory mechanisms vary among bacterial species. In our study, we have shown differential expression of some conserved heat-responsive proteins, such as DnaK and GroEL. However, we have also reported the up-regulation of proteins involved in a variety of metabolic pathways, including translation factors and oxidative stress-responsive proteins, indicating that the responses of *R. tropici* strain PRF 81 to heat stress go beyond the induction of heat-shock proteins.

Strain PRF 81 is known for its high efficiency in fixing nitrogen, and in our study two component response regulators (NtrX and ChvI) were induced during the heat stress. These proteins act in the regulation of the nitrogen-fixation-gene expression and in the regulation of the succinoglycan exopolysaccharide (EPSI) production, respectively, showing that, even under stress conditions, PRF 81 retains nitrogen-fixing and symbiosis-establishment potential, which are essential characteristics for agricultural inoculants.

Finally, this proteomic experiment provides valuable protein-expression information relevant to the ongoing genome sequencing of strain PRF 81 ( http://www.bnf.lncc.br), and contributes to our still-poor knowledge of the molecular determinants of the thermotolerance exhibited by *R. tropici* species. It is a useful reminder that *R. tropici* is an important species of agronomic interest for its capacity to fix nitrogen under tropical stressful conditions, and also demonstrates high resemblance in many genes, and —now also confirmed in many proteins—to those in pathogenic strains of the genus *Agrobacterium*.

## Abbreviations

N: Nitrogen; 2DE: Bidimensional electrophoresis; MALDI: Matrix assisted laser desorption ionization; TOF: Time of flight; COG: Clusters of Orthologous Groups; BNF: Biological nitrogen fixation; TY: Tryptone-yeast extract medium; HCCA: α-cyano-4-hydroxy-cinnamic acid; PMF: Peptide mass fingerprint; MS: Mass spectrometry; EPS: Exopolysaccharides; IEF: Isoelectric focusing; IPG: Immobilized pH gradient; ROS: Reactive oxygen species; Bfr: Bacterioferritin; Bcp: Bacterioferritin comigratory protein.

## Authors’ contributions

MH planned and coordinated the research project. DFG and JSdaSB performed the experiments, analyzed the data and drafted the manuscript. ALS helps in the experiments. DSA and MH contributed to manuscript preparation. All Authors contributed in writing the manuscript and approved its final content.

## Supplementary Material

Additional file 1 **Table S1.** Information about mass spectrometry identification of differentially expressed proteins. All the information contained in Table S1 were obtained for differentially expressed proteins by Mascot (Matrix Science) searches against the public database NCBInr. These spectrometry datasets are also available at PRIDE ( http://ebi.ac.uk/ pride/) with the experiment accession number 14817. Click here for file
