# Supplementary material for: Proteomic profiling of Rhizobium tropici PRF 81: identification of conserved and specific responses to heat stress
Source: BMC Microbiol. 2012 May 30;12:84. doi: 10.1186/1471-2180-12-84 (PMC3502158; doi:10.1186/1471-2180-12-84)
Supplement: Additional file 1 — Table S1. Information about mass spectrometry identification of differentially expressed proteins. All the information contained in Table S1 were obtained for differentially expressed proteins by Mascot (Matrix Science) searches against the public database NCBInr. These spectrometry datasets are also available at PRIDE ( http://ebi.ac.uk/ pride/) with the experiment accession number 14817. [file 1471-2180-12-84-S1.doc]

| **Spot ID** | **Peptide/Protein identification Mascot data** |
| --- | --- |
| 1 | **Identification**: PMF  **GI number:** gi|46909738  **Product:** Isocitrate dehydrogenase [*Rhizobium leguminosarum* bv. *viciae*] **Gene:** *icd*  **Score:** **88 Expect:** 0.0056  **Matches:** 7  **Nominal mass (M_r_):** 45320; **Calculated pI value:** 5.90  Number of mass values searched: **11** Number of mass values matched: **7** Sequence Coverage: **17%**  Start - End Observed Mr(expt) Mr(calc) ppm Miss Sequence  21 - 27 913.7120 912.7047 912.5797 137 0 R.IIWQLIK.D  101 - 109 988.7390 987.7317 987.5865 147 0 R.NILGGVIFR.E  101 - 115 1729.0650 1728.0577 1727.975 48 1 R.NILGGVIFREPIICK.N  120 - 132 1421.9360 1420.9287 1420.8555 52 0 R.LVPGWTKPIVVGR.H  203 - 210 993.6130 992.6057 992.5331 73 0 K.WPVYLSTK.N  259 - 268 1213.6470 1212.6397 1212.5386 83 0 K.WSGGYVWACK.N  320 - 336 1868.9940 1867.9867 1867.9064 43 0 K.GQETSTNSIASIFAWTR.G |
| 2 | **Identification:** PMF  **GI number:** [gi|222087461](http://www.matrixscience.com/cgi/protein_view.pl?file=../data/20100720/FtemiiTam.dat&hit=1)  **Product:** Succinyl-CoA synthetase beta subunit protein [*Agrobacterium radiobacter* K84] **Gene:** *sucC*  **Mass:** 42028    **Score:** **80**  **Expect:** 0.03  **Matches:** 6   Sequence Coverage: **27%**  Nominal mass (M_r_): **42028**; Calculated pI value: **4.98**  Start - End Observed Mr(expt) Mr(calc) ppm Miss Sequence  106 - 116 1279.7740 1278.7667 1278.6092 123 0 R.LYIEDGADIDR.E  117 - 126 1220.8460 1219.8387 1219.6812 129 0 R.ELYLSILVDR.S  127 - 153 2819.4600 2818.4527 2818.3331 42 0 R.SVGQVAFVVSTEGGMDIETVAHDTPEK.I Oxidation (M)  288 - 305 1779.9060 1778.8987 1778.8839 8 0 K.LYGAEPANFLDVGGGATK.E  314 - 334 2186.2330 2185.2257 2185.2181 4 0 K.IITADPAVQGILVNIFGGIMK.C Oxidation (M)  370 - 390 2201.1420 2200.1347 2200.0859 22 0 K.IINESGLNVISADDLDDAAQK.I |
| 3 | **Identification:** PMF  **GI number:** [gi|86359524](http://www.matrixscience.com/cgi/protein_view.pl?file=../data/20100729/FtepfnutE.dat&hit=1)  **Product:** Aconitate hydratase [*Rhizobium etli* CFN 42] **Gene:** *acnA* **EC number:** [4.2.1.3](http://eutils.ncbi.nlm.nih.gov:80/entrez/eutils/"http:/www.expasy.org/enzyme/4.2.1.3")  **Mass:** 97180    **Score:** **87**  **Expect:** 0.0069  **Matches:** 9   Nominal mass (M_r_): **97180**; Calculated pI value: **5.48**  Number of mass values searched: **17** Number of mass values matched: **9** Sequence Coverage: **10%**  Start - End Observed Mr(expt) Mr(calc) ppm Miss Sequence  4 - 11 998.5710 997.5637 997.4287 135 0 K.SLDSFNCR.S  46 - 53 969.7460 968.7387 968.6018 141 0 K.VLLENLLR.F  89 - 107 2033.1540 2032.1467 2032.0486 48 0 R.VLMQDFTGVPAVVDLAAMR.D  148 - 155 1050.6340 1049.6267 1049.5141 107 0 K.NVELEYQR.N  261 - 279 2086.3590 2085.3517 2085.1868 79 1 K.LKEGVTATDLVLTVVQMLR.K  263 - 279 1845.0090 1844.0017 1844.0078 -3 0 K.EGVTATDLVLTVVQMLR.K  525 - 536 1171.7670 1170.7597 1170.6721 75 0 K.GLIVSGVLSGNR.N  588 - 602 1856.9280 1855.9207 1855.9105 6 0 R.DIWPTSHEVQEFIQK.Y  797 - 805 1020.6700 1019.6627 1019.5400 120 0 K.AVIAQSFER.I |
| 4 | **Identification:**  **GI number:** [gi|116254139](http://www.matrixscience.com/cgi/protein_view.pl?file=../data/20100802/FteprfuTh.dat&hit=1)  **Product:** F0F1 ATP synthase subunit beta [*Rhizobium leguminosarum* bv. *viciae* 3841] **Gene:** *atpD*  **Mass:** 50885    **Score:** **86**  **Expect:** 0.0074  **Matches:** 7   decoy: 66 Nominal mass (M_r_): **50885**; Calculated pI value: **5.03**  Number of mass values searched: **30** Number of mass values matched: **7** Sequence Coverage: **22%**  Start - End Observed Mr(expt) Mr(calc) ppm Miss Sequence  178 - 191 1406.5040 1405.4967 1405.6739 -126 0 K.AHGGYSVFAGVGER.T  178 - 193 1663.7100 1662.7027 1662.8227 -72 1 K.AHGGYSVFAGVGERTR.E  218 - 232 1573.5760 1572.5687 1572.7718 -129 0 K.AALVYGQMNEPPGAR.A  263 - 276 1435.5770 1434.5697 1434.7467 -123 0 R.FTQAGSEVSALLGR.I  277 - 297 2293.3450 2292.3377 2292.0878 109 0 R.IPSAVGYQPTLATDMGQMQER.I  385 - 408 2599.4470 2598.4397 2598.3574 32 1 K.ALQDIIAILGMDELSEDDKIAVAR.A  415 - 432 1957.9260 1956.9187 1956.9986 -41 0 R.FLSQPFFVAEVFTGSPGK.L |
| 5 | **Identification:** MS/MS  **GI number:**[gi|1245379](http://www.matrixscience.com/cgi/protein_view.pl?file=../data/20100722/FtemlzTOt.dat&hit=gi|1245379&db_idx=1&px=1&ave_thresh=48&_ignoreionsscorebelow=0&report=0&_sigthreshold=0.05&_msresflags=1025&_msresflags2=2&percolate=-1&percolate_rt=0)  **Product:** Glutamine synthetase I [*Sinorhizobium meliloti*] **Gene:** *glnA*  **Mass:** 52287    **Score:** 81     **Matches:** 1(1)  **Sequences:** 1(1)   Nominal mass (M_r_): **52287**; Calculated pI value: **5.20**  Sequence Coverage: **3%**  Start - End Observed Mr(expt) Mr(calc) ppm Miss Sequence  323 - 338 1718.8460 1717.8387 1717.9403 -59 0 R.LVPGYEAPVLLAYSAR.N ([Ions score 81](http://www.matrixscience.com/cgi/peptide_view.pl?file=../data/20100722/FtemlzTOt.dat&query=1&hit=1&index=gi|1245379&px=1&section=5&ave_thresh=48&_ignoreionsscorebelow=0&report=0&_sigthreshold=0.05&_msresflags=1025&_msresflags2=2&percolate=-1&percolate_rt=0)) |
| 6 | **Identification:** PMF  **GI number:** [gi|15887731](http://www.matrixscience.com/cgi/protein_view.pl?file=../data/20100816/FtepIfstt.dat&hit=1)  **Product:** Acetylglutamate kinase [*Agrobacterium tumefaciens* str. C58] **Gene:** *argB* **EC number:** 2.7.2.8  **Mass:** 31082    **Score:** **72**  **Expect:** 0.00093  **Matches:** 5   Nominal mass (M_r_): **31083**; Calculated pI value: **5.16**  Number of mass values matched: **5** Sequence Coverage: **18%**  Start - End Observed Mr(expt) Mr(calc) ppm Miss Sequence  23 - 31 1093.6200 1092.6127 1092.6179 -5 1 K.YENKTIVVK.Y  94 - 108 1602.8280 1601.8207 1601.8698 -31 0 K.TVEIVEMVLAGSINK.E  94 - 108 1618.8260 1617.8187 1617.8647 -28 0 K.TVEIVEMVLAGSINK.E Oxidation (M)  151 - 165 1645.8400 1644.8327 1644.8723 -24 0 R.VLDLGFVGEVVEVDR.T  174 - 187 1436.7580 1435.7507 1435.7857 -24 0 K.SEMIPVIAPVAPGR.D |
| 7 | **Identification:** PMF  **GI number:** [gi|89258357](http://www.matrixscience.com/cgi/protein_view.pl?file=../data/20100817/FtepIxSem.dat&hit=1)  **Product:** Putative periplasmic substrate binding protein *[Ochrobactrum anthropi*] **Gene:**  **Mass:** 28188    **Score:** **84**  **Expect:** 0.012  **Matches:** 6   Nominal mass (M_r_): **28188**; Calculated pI value: **5.84**  Number of mass values searched: **13** Number of mass values matched: **6** Sequence Coverage: **25%**  Start - End Observed Mr(expt) Mr(calc) ppm Miss Sequence  74 - 93 2211.1040 2210.0967 2210.2245 -58 0 R.MQLKPQLITTAWDGIVAGLR.A  126 - 137 1320.5240 1319.5167 1319.7085 -145 1 R.AVFVKEDSAVQK.L  131 - 142 1360.5350 1359.5277 1359.6882 -118 1 K.EDSAVQKLDDLK.G  145 - 156 1284.6440 1283.6367 1283.6721 -28 0 K.TLGVTLGETHEK.W  160 - 170 1296.6010 1295.5937 1295.6622 -53 1 R.SQGGWTIRTYK.G  226 - 232 842.5090 841.5017 841.4657 43 1 R.KNNPELK.A |
| 8 | **Identification:** PMF  **GI number:** [gi|222109054](http://www.matrixscience.com/cgi/protein_view.pl?file=../data/20100817/FtepIzueT.dat&hit=3)  **Product:** Opine permease ATP-binding protein [*Agrobacterium radiobacter* K84] **Gene:** *nocP*  **Mass:** 28288    **Score:** **86**  **Expect:** 3.8e-05  **Matches:** 6   Nominal mass (M_r_): **28288**; Calculated pI value: **6.98**  Number of mass values searched: **9** Number of mass values matched: **6** Sequence Coverage: **29%**  Start - End Observed Mr(expt) Mr(calc) ppm Miss Sequence  25 - 46 2047.0920 2046.0847 2046.0957 -5 1 K.GISLTANKGDVVSIIGSSGSGK.S  47 - 61 1873.8770 1872.8697 1872.8862 -9 1 K.STFLRCMNFLETPNK.G Oxidation (M)  62 - 74 1383.6750 1382.6677 1382.7882 -87 1 K.GRIAVGQEEVVVK.T  205 - 217 1493.6820 1492.6747 1492.7167 -28 0 R.TMVVVTHEMGFAR.D Oxidation (M)  205 - 222 1993.9770 1992.9697 1992.9761 -3 1 R.TMVVVTHEMGFARDVSSK.V  249 - 257 1052.5540 1051.5467 1051.5484 -2 1 R.CRAFLSSVL.- |
| 9 | **Identification:** PMF  **GI number:** [gi|222087066](http://www.matrixscience.com/cgi/protein_view.pl?file=../data/20100817/FtepIzSwR.dat&hit=1)  **Product:** Oligoendopeptidase F protein [*Agrobacterium radiobacter* K84] **Gene:** *pepF*  **Mass:** 68989    **Score:** **71**  **Expect:** 0.001  **Matches:** 5   Nominal mass (M_r_): **68989**; Calculated pI value: **5.32**  Number of mass values matched: **5** Sequence Coverage: **11%**  Start - End Observed Mr(expt) Mr(calc) ppm Miss Sequence  124 - 140 2033.0390 2032.0317 2032.0782 -23 0 R.LTDYAAHLLFFPLELNR.I  196 - 205 1240.6960 1239.6887 1239.5805 87 0 R.LFDETMAELR.F Oxidation (M)  213 - 230 2124.0700 2123.0627 2123.1296 -32 1 K.LPLEVTLNMLQEKDPEVR.R  361 - 375 1805.8470 1804.8397 1804.8784 -21 0 R.FFDEEWIDAPVRPGK.A  477 - 485 1202.7270 1201.7197 1201.5768 119 0 R.QIAFYEFER.K |
| 10 | **Identification:** PMF  **GI number:** [gi|222087908](http://www.matrixscience.com/cgi/protein_view.pl?file=../data/20100818/FtepCrEeO.dat&hit=1)  **Product:** Aspartate-B-semialdehyde dehydrogenase protein [*Agrobacterium radiobacter* K84]  **Mass:** 37925    **Score:** **80**  **Expect:** 0.00014  **Matches:** 5  ] Nominal mass (M_r_): **37925**; Calculated pI value: **5.46**  Number of mass values searched: **8** Number of mass values matched: **5** Sequence Coverage: **22%**  Start - End Observed Mr(expt) Mr(calc) ppm Miss Sequence  26 - 39 1402.8300 1401.8227 1401.7252 70 0 R.GFPADEVVALASAR.S  100 - 122 2601.2900 2600.2827 2600.3486 -25 1 R.YDADVPLIVPEVNPDAITQFTKR.N  236 - 260 2801.3970 2800.3897 2800.3668 8 0 R.VPVFIGHSESVNIEFENEITADQAR.D  318 - 334 1808.0340 1807.0267 1807.0679 -23 1 R.KGAALNAIQIAELLINR.G  319 - 334 1679.9670 1678.9597 1678.9729 -8 0 K.GAALNAIQIAELLINR.G |
| 11 | **Identification:** PMF  **GI number:** [gi|222084786](http://www.matrixscience.com/cgi/protein_view.pl?file=../data/20100816/FtepIaetm.dat&hit=1)  **Product:** Diaminobutyrate--pyruvate aminotransferase protein [*Agrobacterium radiobacter* K84] **Gene:** *argD*  **Mass:** 42909    **Score:** **84**  **Expect:** 0.012  **Matches:** 6   Nominal mass (M_r_): **42909**; Calculated pI value: **5.63**  Number of mass values matched: **6**  Sequence Coverage: **19%**  Start - End Observed Mr(expt) Mr(calc ) ppm Miss Sequence  2 - 13 1430.8310 1429.8237 1429.6547 118 0 M.AETAPLYDTYMR.A  2 - 13 1446.8130 1445.8057 1445.6497 108 0 M.AETAPLYDTYMR.A Oxidation (M)  92 - 107 1756.9400 1755.9327 1755.8502 47 0 K.VFFTNSGAEALECAIK.T  155 - 170 1703.9340 1702.9267 1702.8315 56 0 K.APGFDQVPFGDLDAVR.A  204 - 224 2424.3710 2423.3637 2423.1937 70 0 R.QICDEHGLLLILDEVQCGVGR.T  365 - 377 1407.9790 1406.9717 1406.8133 113 0 R.LLPPLVVTAEEAR.E |
| 12 | **Identification:** PMF  **GI number:** [gi|114765810](http://www.matrixscience.com/cgi/protein_view.pl?file=../data/20100722/FtemlzsnR.dat&hit=1)  **Product:** Branched-chain amino acid aminotransferase [*Roseovarius sp*. HTCC2601]  **Mass:** 32142    **Score:** **82**  **Expect:** 0.019  **Matches:** 5  Number of mass values searched: **6** Number of mass values matched: **5** Sequence Coverage: **21%** Nominal mass (M_r_): **32142**; Calculated pI value: **5.31**  Start - End Observed Mr(expt) Mr(calc) ppm Miss Sequence  2 - 8 825.2300 824.2227 824.3300 -130 0 M.EGAYDDR.D  83 - 97 1724.8730 1723.8657 1723.8893 -14 1 K.DEVLKVNGFTNAYVR.A  115 - 134 2251.8930 2250.8857 2251.069 -82 0 R.NPVQLAVAAWEWGNYYGDAK.T  147 - 158 1331.6810 1330.6737 1330.7357 -47 0 K.RPSPETIPVHAK.A  221 - 228 970.5580 969.5507 969.6335 -85 1 R.QTVIRLLK.E |
| 13 | **Identification:** PMF  **GI number:** [gi|86146888](http://www.matrixscience.com/cgi/protein_view.pl?file=../data/20100816/FtepIfaaS.dat&hit=1)  **Product:** Uridylate kinase [*Vibrio sp.* MED222]  **Mass:** 26284    **Score:** **86**  **Expect:** 0.0086  **Matches:** 6   Nominal mass (M_r_): **26284**; Calculated pI value: **5.08**  Number of mass values searched: **7** Number of mass values matched: **6** Sequence Coverage: **23%**  Start - End Observed Mr(expt) Mr(calc) ppm Miss Sequence  43 - 62 2055.2040 2054.1967 2054.1525 22 0 K.ELVELGVQVGVVIGGGNLFR.G  63 - 73 1062.6580 1061.6507 1061.4924 149 0 R.GAGLAEAGMNR.V Oxidation (M)  199 - 209 1223.7190 1222.7117 1222.6380 60 0 K.VMDLAAFTLAR.D Oxidation (M)  199 - 212 1587.9510 1586.9437 1586.8239 76 1 K.VMDLAAFTLARDHK.M  199 - 212 1603.9790 1602.9717 1602.8188 95 1 K.VMDLAAFTLARDHK.M Oxidation (M)  217 - 228 1402.7090 1401.7017 1401.7663 -46 1 R.VFNMNKPGALRR.V |
| 14 | **Identification:** PMF  **GI number:** [gi|222085874](http://www.matrixscience.com/cgi/protein_view.pl?file=../data/20100720/Ftemixswe.dat&hit=1)  **Product:** Phosphopyruvate hydratase [*Agrobacterium radiobacter* K84] **Gene:** *eno*  **Mass:** 45120    **Score:** **86**  **Expect:** 0.0089  **Matches:** 7 Nominal mass (M_r_): **45120**; Calculated pI value: **4.84**  Sequence Coverage: **22%**  Start - End Observed Mr(expt) Mr(calc) ppm Miss Sequence  35 - 52 1722.9880 1721.9807 1721.8696 65 0 R.AAVPSGASTGAHEAVELR.D  120 - 131 1274.8050 1273.7977 1273.6778 94 0 K.AAAQSANLPLYR.Y  278 - 296 2240.0570 2239.0497 2238.9779 32 0 K.YPIVSIEDGMAEDDWEGWK.A  278 - 296 2256.1620 2255.1547 2254.9729 81 0 K.YPIVSIEDGMAEDDWEGWK.A Oxidation (M)  297 - 304 830.6190 829.6117 829.4909 146 0 K.ALTDLIGK.K  306 - 320 1635.9130 1634.9057 1634.8264 49 0 K.TQLVGDDLFVTNSAR.L  366 - 387 2280.1430 2279.1357 2279.0587 34 0 R.SGETEDSTIADLAVATNCGQIK.T |
| 15 | **Identification:** PMF  **GI number:** [gi|282887091](http://www.matrixscience.com/cgi/protein_view.pl?file=../data/20100722/FtemlzewL.dat&hit=1)  **Product:** Alpha amylase catalytic region [*Burkholderia* sp. CCGE1001]  **Mass:** 64245    **Score:** **78**  **Expect:** 0.052  **Matches:** 7   Nominal mass (M_r_): **64245**; Calculated pI value: **6.26**  Number of mass values searched: **8** Number of mass values matched: **7** Sequence Coverage: **14%**  Start - End Observed Mr(expt) Mr(calc) ppm Miss Sequence  61 - 75 1777.8640 1776.8567 1776.7479 61 0 R.DNGYDISDYYNVDPK.Y  76 - 90 1760.8360 1759.8287 1759.7624 38 0 K.YGTLGDFSEFTHACR.E  149 - 158 1212.6500 1211.6427 1211.5822 50 1 K.STWTFDKEAK.R  375 - 390 1835.9640 1834.9567 1834.8519 57 1 R.YGDEIGMGDDLRLPER.E  375 - 390 1851.9750 1850.9677 1850.8469 65 1 R.YGDEIGMGDDLRLPER.E Oxidation (M)  436 - 449 1807.0080 1806.0007 1805.8552 81 1 R.DPNSLLNWMERMIR.M 2 Oxidation (M)  542 - 550 1005.6150 1004.6077 1004.5655 42 0 R.VGGLDYLLR.R |
| 16 | **Identification:** PMF  **GI number:** [gi|241206422](http://www.matrixscience.com/cgi/protein_view.pl?file=../data/20100723/FtemIrste.dat&hit=1)  **Product: T**ransaldolase [*Rhizobium leguminosarum* bv. *trifolii* WSM1325]  **Mass:** 35091    **Score:** **82**  **Expect:** 0.02  **Matches:** 6   Nominal mass (M_r_): **35091**; Calculated pI value: **5.32**  Number of mass values searched: **9** Number of mass values matched: **6** Sequence Coverage: **23%**  Start - End Observed Mr(expt) Mr(calc) ppm Miss Sequence  77 - 92 1564.0670 1563.0597 1562.9872 46 1 R.LAISVGAALVKLVPGR.V  172 - 187 1952.9740 1951.9667 1952.0924 -64 1 K.VFLISPFVGRILDWYK.K  193 - 206 1576.6660 1575.6587 1575.7417 -53 0 K.DYTPEEDPGVISVR.E  214 - 227 1522.8820 1521.8747 1521.7973 51 1 K.ANDIKTIVMGASFR.S  294 - 303 1075.6950 1074.6877 1074.6185 64 1 K.LAEGIRAFAK.D  300 - 309 1091.6930 1090.6857 1090.6135 66 1 R.AFAKDLGTLR.T |
| 17 | **Identification:** PMF  **GI number:** [gi|11493200](http://www.matrixscience.com/cgi/protein_view.pl?file=../data/20100729/FtepfGSmR.dat&hit=1)  **Product:** Phosphoglucomutase [Rhizobium tropici] **Gene:** *pgm*  **Mass:** 58641    **Score:** **82**  **Expect:** 0.02  **Matches:** 7   Nominal mass (M_r_): **58641**; Calculated pI value: **5.16**  Number of mass values searched: **11** Number of mass values matched: **7** Sequence Coverage: **13%**  Start - End Observed Mr(expt) Mr(calc) ppm Miss Sequence  86 - 99 1369.6710 1368.6637 1368.7725 -79 0 K.GGILSTPAASNIIR.K  138 - 145 980.4930 979.4857 979.4974 -12 0 K.ITDAIYER.S  199 - 206 919.5440 918.5367 918.5287 9 0 R.NLISLGFR.I  222 - 227 806.4670 805.4597 805.4334 33 0 K.EIFEIR.L  335 - 346 1307.5400 1306.5327 1306.6558 -94 0 R.GVGIYETPTGWK.F  392 - 408 1931.6900 1930.6827 1930.9537 -140 0 R.GESVIDIVTQHWATYGR.N  463 - 471 1011.5150 1010.5077 1010.5257 -18 0 K.SVSQHQGIR.I |
| 18 | **Identification:** PMF  **GI number:** [gi|222084905](http://www.matrixscience.com/cgi/protein_view.pl?file=../data/20100817/FtepIbuSh.dat&hit=1)  **Product:** Alpha-glucosidase protein [*Agrobacterium radiobacter* K84]  **Mass:** 62592    **Score:** **78**  **Expect:** 0.00024  **Matches:** 5   Nominal mass (M_r_): **62592**; Calculated pI value: **4.84**  Number of mass values matched: **5** Sequence Coverage: **14%**  Start - End Observed Mr(expt) Mr(calc) ppm Miss Sequence  1 - 17 1993.9200 1992.9127 1992.9727 -30 1 -.MSIASQSIATVDKDWWR.G  105 - 117 1487.7820 1486.7747 1486.7450 20 0 K.VMIDLVLSHSSDR.H Oxidation (M)  266 - 285 2059.9960 2058.9887 2059.0222 -16 0 R.AVLDEYPAIAAVGEVGDSQR.G  446 - 463 2078.0060 2076.9987 2077.0188 -10 1 R.AVSVQQGDENSVLEQYRR.F  537 - 548 1379.7320 1378.7247 1378.7034 16 0 K.IDIPAWGAYFAR.L |
| 19 | **Identification:** MS/MS  **GI number:** [gi|222086485](http://www.matrixscience.com/cgi/protein_view.pl?file=../data/20100722/FtemlxYEh.dat&hit=gi|222086485&db_idx=1&px=1&ave_thresh=46&_ignoreionsscorebelow=0&report=0&_sigthreshold=0.05&_msresflags=1025&_msresflags2=2&percolate=-1&percolate_rt=0)  **Product:** ABC transporter [Agrobacterium radiobacter K84]  Pep. Mass: 1296.630    **Mass:** 38975    **Score:** 89     **Matches:** 1(1)  **Sequences:** 1(1)  Nominal mass (M_r_): **38975**; Calculated pI value: **5.23**  Sequence Coverage: **5%**  Start - End Observed Mr(expt) Mr(calc) ppm Miss Sequence  64 - 85 2346.9930 2345.9857 2346.0975 -48 0 K.YGIEVNELNPDAGSGDEVEAIR.A ([Ions score 89](http://www.matrixscience.com/cgi/peptide_view.pl?file=../data/20100722/FtemlxYEh.dat&query=1&hit=1&index=gi|222086485&px=1&section=5&ave_thresh=46&_ignoreionsscorebelow=0&report=0&_sigthreshold=0.05&_msresflags=1025&_msresflags2=2&percolate=-1&percolate_rt=0)) |
| 20 | **Identification:** PMF  **GI number:**[gi|296105270](http://www.matrixscience.com/cgi/protein_view.pl?file=../data/20100726/FtemCGEah.dat&hit=1)  **Product:** Biotin--protein ligase [Enterobacter cloacae subsp. cloacae ATCC 13047] **Mass:** 35255    **Score:** **80**  **Expect:** 0.03  **Matches:** 5   Nominal mass (M_r_): **35255**; Calculated pI value: **5.23**  Number of mass values searched: **7** Number of mass values matched: **5** Sequence Coverage: **29%**  Start - End Observed Mr(expt) Mr(calc) ppm Miss Sequence  34 - 44 1264.8240 1263.8167 1263.7411 60 1 R.AAINKHIQTLR.D  184 - 212 2927.2110 2926.2037 2926.5984 -135 1 K.LAGILVELTGKTGDAAQIVIGAGLNMVMR.N Oxidation (M)  245 - 264 2283.1800 2282.1727 2282.1906 -8 1 K.ELRTSLSLFEQEGLASFLSR.W  248 - 267 2328.1890 2327.1817 2327.1797 1 1 R.TSLSLFEQEGLASFLSRWEK.L  291 - 320 3122.0840 3121.0767 3120.6455 138 1 R.GIDAQGALLLEQDGVIKPWVGGEISLRSAE.- |
| 21 | **Identification:** PMF  **GI number:** [gi|299768808](http://www.matrixscience.com/cgi/protein_view.pl?file=../data/20100802/FteprfHOm.dat&hit=1)  **Product:** Acyl-CoA dehydrogenase [*Acinetobacter* sp. DR1]  **Mass:** 66035    **Score:** **84**  **Expect:** 0.012  **Matches:** 7   Nominal mass (M_r_): **65994**; Calculated pI value: **5.37**  Number of mass values searched: **14** Number of mass values matched: **7** Sequence Coverage: **15%**  Start - End Observed Mr(expt) Mr(calc) ppm Miss Sequence  259 - 275 1875.9030 1874.8957 1874.8767 10 0 K.MGIHGNATCVINFDQAK.G  327 - 336 1013.4730 1012.4657 1012.5553 -88 1 R.SLSGPKAPEK.E  333 - 348 1741.9640 1740.9567 1740.9522 3 1 K.APEKEADPIIVHPAVR.N  349 - 362 1535.8230 1534.8157 1534.7926 15 1 R.NMLLTQKAFAEGGR.A  400 - 411 1240.6240 1239.6167 1239.5983 15 0 K.AFLTETGSESAK.H  498 - 509 1436.6520 1435.6447 1435.6588 -10 0 K.EWGDLTMQIGMR.A  548 - 561 1543.9070 1542.8997 1542.7202 116 0 K.LAEGTTDVDFYNAK.V |
| 22 | **Identification:** MS/MS  **GI number:** [gi|282888281](http://www.matrixscience.com/cgi/protein_view.pl?file=../data/20100816/FtepIfcaO.dat&hit=gi|282888281&db_idx=1&px=1&ave_thresh=47&_ignoreionsscorebelow=0&report=0&_sigthreshold=0.05&_msresflags=1025&_msresflags2=2&percolate=-1&percolate_rt=0)  **Product:** 3-Oxoacyl-(acyl-carrier-protein (ACP)) synthase III domain protein [Burkholderia sp. CCGE1001]  **Mass:** 38552    **Score:** 51     **Matches:** 1(1)  **Sequences:** 1(1)   Nominal mass (M_r_): **38552**; Calculated pI value: **6.27**  Sequence Coverage: **6%**  Start - End Observed Mr(expt) Mr(calc) ppm Miss Sequence  5 - 25 2285.0470 2284.0397 2284.1845 -63 1 K.NVAVSLPSRVVTNDEVADMIR.F ([Ions score 51](http://www.matrixscience.com/cgi/peptide_view.pl?file=../data/20100816/FtepIfcaO.dat&query=1&hit=1&index=gi|282888281&px=1&section=5&ave_thresh=47&_ignoreionsscorebelow=0&report=0&_sigthreshold=0.05&_msresflags=1025&_msresflags2=2&percolate=-1&percolate_rt=0)) |
| 23 | **Identification:** PMF  **GI number:** [gi|159186213](http://www.matrixscience.com/cgi/protein_view.pl?file=../data/20100818/FtepCrHmE.dat&hit=1)  **Product:** Beta-ketoadipyl CoA thiolase [*Agrobacterium tumefaciens* str. C58] **Gene:** *pcaF*  **Mass:** 41850    **Score:** **80**  **Expect:** 0.00014  **Matches:** 5   Nominal mass (M_r_): **41850**; Calculated pI value: **5.51**  Number of mass values matched: **5** Sequence Coverage: **13%**  Start - End Observed Mr(expt) Mr(calc) ppm Miss Sequence  89 - 102 1391.7850 1390.7777 1390.6697 78 0 R.LCGSGMDAVIAAAR.A  137 - 148 1438.7920 1437.7847 1437.6888 67 0 R.NAEIYDTTIGWR.F  179 - 187 1050.6240 1049.6167 1049.4778 132 0 R.EDQDAFAVR.S  273 - 281 1018.7370 1017.7297 1017.5971 130 1 R.KYGLTPIAR.I  282 - 293 1078.7640 1077.7567 1077.6295 118 0 R.ILGGAAAGVPPR.V |
| 24 | **Identification:** PMF  **GI number:** [gi|222087891](http://www.matrixscience.com/cgi/protein_view.pl?file=../data/20100727/FtemCzHwt.dat&hit=1)  **Product:** Bacterioferritin [Agrobacterium radiobacter K84] **Gene:** *bfr*  **Mass:** 16860    **Score:** **80**  **Expect:** 0.034  **Matches:** 7   Nominal mass (M_r_): **16860**; Calculated pI value: **4.81**  Number of mass values searched: **16** Number of mass values matched: **7** Sequence Coverage: **37%**  Start - End Observed Mr(expt) Mr(calc) ppm Miss Sequence  17 - 25 1124.5870 1123.5797 1123.5550 22 0 R.LLEDWGYTK.L  30 - 43 1700.8720 1699.8647 1699.7583 63 1 K.ERAESIEEMQHADR.L  30 - 43 1716.8520 1715.8447 1715.7533 53 1 K.ERAESIEEMQHADR.L Oxidation (M)  32 - 43 1415.6710 1414.6637 1414.6147 35 0 R.AESIEEMQHADR.L  32 - 43 1431.6430 1430.6357 1430.6096 18 0 R.AESIEEMQHADR.L Oxidation (M)  48 - 64 1932.2140 1931.2067 1931.0992 56 0 R.IIFLEGHPNLQTLAPLR.I  90 - 104 1753.8760 1752.8687 1752.7560 64 1 K.SRDICHDAGDYVSMK.L |
| 25 | **Identification:** PMF  **GI number:** [gi|87199081](http://www.matrixscience.com/cgi/protein_view.pl?file=../data/20100729/FtepfGTTL.dat&hit=1)  **Product:** TonB-dependent receptor [Novosphingobium aromaticivorans DSM 12444] **Mass:** 87810    **Score:** **82**  **Expect:** 0.021  **Matches:** 7   Nominal mass (M_r_): **87810**; Calculated pI value: **5.82**  Number of mass values searched: **15** Number of mass values matched: **7** Sequence Coverage: **13%**  Start - End Observed Mr(expt) Mr(calc) ppm Miss Sequence  114 - 132 2259.3720 2258.3647 2258.2059 70 1 R.VEILPEEVALKFGYRPDQR.V  125 - 139 1851.9630 1850.9557 1851.0155 -32 1 K.FGYRPDQRVVNFILK.D  223 - 248 2508.4960 2507.4887 2507.3932 38 1 K.SAQVNATLARALGGGAGLSVNLLAQR.D  411 - 427 1775.8920 1774.8847 1774.9214 -21 1 R.GDAQAGFSLDLPITSRK.E  481 - 504 2523.3980 2522.3907 2522.2653 50 0 K.APGLTDLGGPTTVTQNVSLYDFTR.G  601 - 619 2108.2950 2107.2877 2107.0633 107 1 R.LRYGVNLSGSFGKPDPNMR.R  603 - 619 1838.9700 1837.9627 1837.8781 46 0 R.YGVNLSGSFGKPDPNMR.R |
| 26 | **Identification:** PMF  **GI number:** [gi|222086436](http://www.matrixscience.com/cgi/protein_view.pl?file=../data/20100729/FtepfGeEt.dat&hit=1)  **Product:** Cell division protein [*Agrobacterium radiobacter* K84] **Gene:** *ftsZ*2  **Mass:** 63014    **Score:** **100**  **Expect:** 0.00033  **Matches:** 9   Nominal mass (M_r_): **63014**; Calculated pI value: **5.21**  Number of mass values searched: **21** Number of mass values matched: **9** Sequence Coverage: **20%**  Start - End Observed Mr(expt) Mr(calc) ppm Miss Sequence  61 - 82 2194.3190 2193.3117 2193.1754 62 0 R.IIQLGVNVTEGLGAGSQPEVGR.A  128 - 145 1914.1710 1913.1637 1913.0524 58 0 K.GILTVGVVTKPFHFEGGR.R  160 - 174 1729.0630 1728.0557 1727.9570 57 0 K.SVDTLIVIPNQNLFR.I  207 - 218 1361.7650 1360.7577 1360.6987 43 0 K.EGLINLDFADVR.S  260 - 272 1242.7640 1241.7567 1241.7092 38 0 K.GAQGLLISITGGR.D  273 - 285 1479.8080 1478.8007 1478.7253 51 0 R.DLTLFEVDEAATR.I  431 - 442 1269.7380 1268.7307 1268.6877 34 0 R.IFAAAPEAQPVR.Q  542 - 553 1258.6810 1257.6737 1257.6353 31 0 R.APSPEASLYAPR.R  542 - 554 1414.8060 1413.7987 1413.7364 44 1 R.APSPEASLYAPRR.G |
| 27 | **Identification:** PMF  **GI number:** [gi|50121473](http://www.matrixscience.com/cgi/protein_view.pl?file=../data/20100802/Fteprfuem.dat&hit=1)  **Product:** Condesin subunit F [*Pectobacterium atrosepticum* SCRI1043] **Gene:** *kicB*  **Mass:** 50717    **Score:** **83**  **Expect:** 0.015  **Matches:** 6 Nominal mass (M_r_): **50717**; Calculated pI value: **4.70**  Number of mass values searched: **8** Number of mass values matched: **6** Sequence Coverage: **17%**  Start - End Observed Mr(expt) Mr(calc) ppm Miss Sequence  17 - 28 1406.7960 1405.7887 1405.7201 49 1 R.KNDFSISLPTER.L  29 - 42 1519.8150 1518.8077 1518.8228 -10 0 R.LAFLMAIATLNGER.M  125 - 138 1590.8740 1589.8667 1589.8447 14 0 R.LSMQLSIVAQELSR.A Oxidation (M)  302 - 323 2599.2120 2598.2047 2598.2615 -22 1 R.LRQSVQSYFDNPWALTFANADR.L  370 - 384 1791.0320 1790.0247 1789.9396 48 1 K.YKAQQIPLDLSEVMR.E  372 - 384 1515.8460 1514.8387 1514.7763 41 0 K.AQQIPLDLSEVMR.E Oxidation (M) |
| 28 | **Identification:** PMF  **GI number:** [gi|117926246](http://www.matrixscience.com/cgi/protein_view.pl?file=../data/20100727/FtemCzTmt.dat&hit=1)  **Product:** Protein tyrosine phosphatase [*Magnetococcus* sp. MC-1]  **Mass:** 18731    **Score:** **85**  **Expect:** 0.0098  **Matches:** 5   Nominal mass (M_r_): **18731**; Calculated pI value: **6.29**  Number of mass values searched: **8** Number of mass values matched: **5** Sequence Coverage: **35%**  Start - End Observed Mr(expt) Mr(calc) ppm Miss Sequence  20 - 29 1133.7610 1132.7537 1132.5910 144 0 R.SLMAEAILNR.E Oxidation (M)  59 - 69 1296.7930 1295.7857 1295.7020 65 0 K.MHLDTSVLRPK.S  70 - 91 2499.2510 2498.2437 2498.1828 24 0 K.SWSAFIESAPPPIDFAFTLCDK.T  134 - 149 1935.9060 1934.8987 1935.0400 -73 1 R.MITNRISVFVNLPFER.L  139 - 149 1320.7000 1319.6927 1319.7238 -24 0 R.ISVFVNLPFER.L |
| 29 | **Identification:** PMF  **GI number:** [gi|222087232](http://www.matrixscience.com/cgi/protein_view.pl?file=../data/20100817/FtepIzSES.dat&hit=1)  **Product:** Serine protein kinase protein [*Agrobacterium radiobacter* K84]  **Mass:** 74417    **Score:** **122**  **Expect:** 2e-06  **Matches:** 12   Nominal mass (M_r_): **74417**; Calculated pI value: **5.42**  Number of mass values searched: **17** Number of mass values matched: **12** Sequence Coverage: **20%**  Start - End Observed Mr(expt) Mr(calc) ppm Miss Sequence  2 - 13 1397.7210 1396.7137 1396.6735 29 0 M.SNNDVLFNTFAR.S  75 - 91 1965.9250 1964.9177 1964.8978 10 0 R.TYPAFAGFFGMEETIER.I  187 - 197 1350.7030 1349.6957 1349.6575 28 1 K.RLEEFEGDISR.F  188 - 197 1194.6110 1193.6037 1193.5564 40 0 R.LEEFEGDISR.F  237 - 257 2312.0600 2311.0527 2311.1080 -24 1 R.KLESLAQNDPDAYSYSGALNR.A  333 - 338 807.4210 806.4137 806.4109 4 0 K.VPYCLR.V  349 - 370 2402.1950 2401.1877 2401.1682 8 0 K.LIEESELSDAPCAPSTLETLAR.F  483 - 493 1334.7320 1333.7247 1333.6666 44 0 R.YAEFIGHEIQK.A  494 - 509 1924.8650 1923.8577 1923.8639 -3 0 K.AYLESYADYGQNLFDR.Y  617 - 623 873.4370 872.4297 872.4141 18 0 K.HGEFVER.M  637 - 643 996.5300 995.5227 995.4899 33 0 R.LVEWYMR.V  637 - 643 1012.5220 1011.5147 1011.4848 30 0 R.LVEWYMR.V Oxidation (M) |
| 30 | **Identification:** PMF  **GI number:** [gi|116252038](http://www.matrixscience.com/cgi/protein_view.pl?file=../data/20100802/FteprfStE.dat&hit=3)  **Product:** Putative two component response regulator nitrogen assimilation regulatory protein [*Rhizobium leguminosarum* bv. *viciae* 3841]  **Gene:** *ntrX*  **Mass:** 50611    **Score:** **81**  **Expect:** 0.026  **Matches:** 7   Nominal mass (M_r_): **50611**; Calculated pI value: **6.63**  Number of mass values searched: **8** Number of mass values matched: **7** Sequence Coverage: **20%**  Start - End Observed Mr(expt) Mr(calc) ppm Miss Sequence  207 - 223 1791.7460 1790.7387 1790.8985 -89 1 R.MEVALFGTEGTPGQARK.I  233 - 245 1507.7180 1506.7107 1506.7388 -19 0 R.GILYLDEVGEMPR.E Oxidation (M)  254 - 267 1561.7600 1560.7527 1560.8260 -47 1 R.VLVDQQFERVGGSK.R  315 - 326 1475.7550 1474.7477 1474.7490 -1 0 R.EDIPFLVDQLMR.Q  372 - 394 2383.9430 2382.9357 2383.1287 -81 0 R.TDGPDAPITADMLPTDLGDMLPK.V  434 - 447 1603.8210 1602.8137 1602.7936 13 1 R.TAEFVGMERSALHR.K  434 - 447 1619.8150 1618.8077 1618.7886 12 1 R.TAEFVGMERSALHR.K Oxidation (M) |
| 31 | **Identification:** PMF  **GI number:** [gi|159184131](http://www.matrixscience.com/cgi/protein_view.pl?file=../data/20100816/FtepIfsOm.dat&hit=1)  **Product:** Two component response regulator [*Agrobacterium tumefaciens* str. C58] **Gene:** *chvI*  **Mass:** 27253    **Score:** **74**  **Expect:** 0.00048  **Matches:** 4 Nominal mass (M_r_): **27253**; Calculated pI value: **5.56**  Number of mass values matched: **4** Sequence Coverage: **32%**  Start - End Observed Mr(expt) Mr(calc) ppm Miss Sequence  71 - 92 2508.3350 2507.3277 2507.3047 9 1 K.SDIPVIFLTSKDEEIDELFGLK.M  93 - 106 1628.8240 1627.8167 1627.7664 31 0 K.MGADDFITKPFSQR.L Oxidation (M)  161 - 185 2704.6420 2703.6347 2703.5323 38 0 K.GEPVTLTVTEFLILHSLAQRPGVVK.S  188 - 204 2004.8420 2003.8347 2003.8418 -4 0 R.DALMDAAYDEQVYVDDR.T Oxidation (M) |
| 32 | **Identification:** PMF  **GI number:** [gi|222087564](http://www.matrixscience.com/cgi/protein_view.pl?file=../data/20100816/FtepIaYat.dat&hit=1)  **Product:** Thioredoxin [*Agrobacterium radiobacter* K84] **Gene:** *trxA*  **Mass:** 34469    **Score:** **83**  **Expect:** 6.5e-05  **Matches:** 5   Nominal mass (M_r_): **34469**; Calculated pI value: **4.83**  Number of mass values searched: **7** Number of mass values matched: **5** Sequence Coverage: **15%**  Start - End Observed Mr(expt) Mr(calc) ppm Miss Sequence  37 - 52 1796.9340 1795.9267 1795.8588 38 1 K.DTTTANFAKDVIEESR.N  238 - 259 2444.4350 2443.4277 2443.2091 89 1 R.KLGDPVALEQELAANPDNHEQR.V  239 - 259 2316.3170 2315.3097 2315.1142 84 0 K.LGDPVALEQELAANPDNHEQR.V  296 - 308 1649.0000 1647.9927 1647.8773 70 1 R.RQLLEFFEVWGPK.D  297 - 308 1492.9140 1491.9067 1491.7762 87 0 R.QLLEFFEVWGPK.D |
| 33 | **Identification:** PMF  **GI number:** [gi|118590060](http://www.matrixscience.com/cgi/protein_view.pl?file=../data/20100817/FtepIxSnh.dat&hit=1)  **Product:** Bacterioferritin comigratory protein [*Stappia aggregata* IAM 12614]  **Mass:** 16749    **Score:** **81**  **Expect:** 0.023  **Matches:** 6   Nominal mass (M_r_): **16749**; Calculated pI value: **5.63**  Number of mass values searched: **11** Number of mass values matched: **6** Sequence Coverage: **33%**  Start - End Observed Mr(expt) Mr(calc) ppm Miss Sequence  31 - 41 1296.7780 1295.7707 1295.7278 33 0 K.GKPVVVYFYPK.D  42 - 49 893.2610 892.2537 892.3597 -119 0 K.DDTPGCTK.E  82 - 91 1182.7740 1181.7667 1181.7033 54 1 K.FIAKHNLAIR.L  99 - 115 1917.9170 1916.9097 1916.8978 6 1 K.EAAEAYGVWVEKSMYGK.T  116 - 122 855.3040 854.2967 854.3956 -116 0 K.TYMGVER.S  116 - 122 871.2740 870.2667 870.3905 -142 0 K.TYMGVER.S Oxidation (M) |
| 34 | **Identification:** PMF  **GI number:** [gi|58826564](http://www.matrixscience.com/cgi/protein_view.pl?file=../data/20100729/FtemOeSTS.dat&hit=1)  **Product:** DnaK [*Rhizobium tropici*] **Gene:** *dnaK*  **Mass:** 68393    **Score:** **80**  **Expect:** 0.029  **Matches:** 7   Nominal mass (M_r_): **68393**; Calculated pI value: **4.91**  Number of mass values searched: **8** Number of mass values matched: **7** Sequence Coverage: **14%**  Start - End Observed Mr(expt) Mr(calc) ppm Miss Sequence  138 - 151 1564.9870 1563.9797 1563.8045 112 0 K.AVITVPAYFNDAQR.Q  231 - 241 1338.9280 1337.9207 1337.7595 121 1 R.LVEYLVAEFKK.D  298 - 310 1485.9540 1484.9467 1484.8198 85 1 R.AKLESLVDDLVQR.T  321 - 340 1989.1740 1988.1667 1987.9885 90 0 K.DAGVTAAEIDEVVLVGGMSR.M  321 - 340 2005.1500 2004.1427 2003.9834 80 0 K.DAGVTAAEIDEVVLVGGMSR.MOxidationM)  418 - 434 1841.0080 1840.0007 1839.8599 77 0 K.SQTFSTADDNQSAVTIR.V  448 - 462 1593.1270 1592.1197 1591.9086 133 0 K.LLGQFDLVGLPPAPR.G |
| 35 | **Identification:** MS/MS  **GI number:** [gi|222085003](http://www.matrixscience.com/cgi/protein_view.pl?file=../data/20100721/FtemlrewE.dat&hit=gi|222085003&db_idx=1&px=1&ave_thresh=46&_ignoreionsscorebelow=0&report=0&_sigthreshold=0.05&_msresflags=1025&_msresflags2=2&percolate=-1&percolate_rt=0)  **Product:** Chaperonin GroEL [*Agrobacterium radiobacter* K84] **Gene:** *groEL*  **Pep. Mass: 1609.044 -**    **Mass:** 57836    **Score:** 72     **Matches:** 1(1)  **Sequences:** 1(1)   Nominal mass (M_r_): **57836**; Calculated pI value: **5.03**  Sequence Coverage: **3%**  Start - End Observed Mr(expt) Mr(calc) ppm Miss Sequence  405 - 421 1609.0440 1608.0367 1607.8995 85 0 R.AAVQEGIVPGGGTALLR.S ([Ions score 72](http://www.matrixscience.com/cgi/peptide_view.pl?file=../data/20100721/FtemlrewE.dat&query=1&hit=1&index=gi|222085003&px=1&section=5&ave_thresh=46&_ignoreionsscorebelow=0&report=0&_sigthreshold=0.05&_msresflags=1025&_msresflags2=2&percolate=-1&percolate_rt=0)) |
| 36 | **Identification:** PMF  **GI number:** [gi|86359655](http://www.matrixscience.com/cgi/protein_view.pl?file=../data/20100802/FteprisTS.dat&hit=1)  **Product:** Putative metalloendopeptidase protein [*Rhizobium etli* CFN 42]  **Mass:** 49514    **Score:** **84**  **Expect:** 0.011  **Matches:** 7   Nominal mass (M_r_): **49514**; Calculated pI value: **5.36**  Number of mass values searched: **16** Number of mass values matched: **7** Sequence Coverage: **15%**  Start - End Observed Mr(expt) Mr(calc) ppm Miss Sequence  91 - 105 1602.8080 1601.8007 1601.8485 -30 1 K.STQSVRQALIDSAAR.R  151 - 171 2189.9530 2188.9457 2189.1627 -99 1 R.MGRNPPPALLVTPDDALASVR.S  154 - 171 1845.8340 1844.8267 1844.9996 -94 0 R.NPPPALLVTPDDALASVR.S  289 - 296 1030.5410 1029.5337 1029.5567 -22 1 R.RLLTDEQR.A  407 - 420 1436.6590 1435.6517 1435.7534 -71 0 K.FVFAGEPLAVMGAK.R  407 - 421 1592.7470 1591.7397 1591.8545 -72 1 K.FVFAGEPLAVMGAKR.V  444 - 457 1642.8480 1641.8407 1641.8263 9 0 K.DGKPVDSRPWWTAK.D |
| 37 | **Identification:** PMF  **GI number:** [gi|222085864](http://www.matrixscience.com/cgi/protein_view.pl?file=../data/20100729/FtepfnSOe.dat&hit=1)  **Product:** Outer membrane lipoprotein [*Agrobacterium radiobacter* K84] **Gene:** *omp*1  **Mass:** 84589    **Score:** **84**  **Expect:** 0.013  **Matches:** 12   Nominal mass (M_r_): **84589**; Calculated pI value: **5.26**  Number of mass values searched: **36** Number of mass values matched: **12** Sequence Coverage: **19%**  Start - End Observed Mr(expt) Mr(calc) ppm Miss Sequence  175 - 186 1346.7380 1345.7307 1345.6990 24 0 R.INVAFVINEGDR.T  231 - 239 1044.5790 1043.5717 1043.4996 69 0 R.QQADQDALR.Q  240 - 251 1580.7140 1579.7067 1579.6844 14 0 R.QFYYNHGYADFR.I  325 - 334 1038.6030 1037.5957 1037.5294 64 0 R.VASAGYPFAR.I  342 - 359 2001.9910 2000.9837 2000.9803 2 0 R.DLSGHTIGIEYLVDQGER.A  401 - 410 1183.7060 1182.6987 1182.6397 50 1 R.RLDALGYFTK.V  483 - 497 1870.9120 1869.9047 1869.8937 6 0 R.TYNISFTEPYFLGYR.L  498 - 506 981.6070 980.5997 980.5331 68 0 R.LAAGFDIFK.N  526 - 539 1499.8660 1498.8587 1498.8355 15 0 R.VTAPITENLATTLR.Y  648 - 659 1366.7520 1365.7447 1365.7041 30 0 K.LNVFDQFTLGGR.E  663 - 672 1017.5830 1016.5757 1016.5039 71 0 R.GFENAGIGPR.T  735 - 751 1730.9560 1729.9487 1729.9515 -2 0 R.ASLGAGLIWSSPFGVIR.V |
| 38 | **Identification:** PMF  **GI number:** [gi|18033179](http://www.matrixscience.com/cgi/protein_view.pl?file=../data/20100817/FtepIbame.dat&hit=1)  **Product:** VirD4 [*Agrobacterium tumefaciens*]  **Mass:** 73380    **Score:** **80**  **Expect:** 0.00013  **Matches:** 8   Nominal mass (M_r_): **73380**; Calculated pI value: **6.82**  Number of mass values searched: **15** Number of mass values matched: **8** Sequence Coverage: **17%**  Start - End Observed Mr(expt) Mr(calc) ppm Miss Sequence  89 - 97 1064.5200 1063.5127 1063.4908 21 1 R.DRDHHGTAR.W  91 - 105 1683.8850 1682.8777 1682.7332 86 1 R.DHHGTARWAGSGEMR.H Oxidation (M)  113 - 128 1767.8340 1766.8267 1766.8774 -29 1 R.YSQVTGPIFGKTCGPR.W  187 - 202 1751.8160 1750.8087 1750.8526 -25 1 K.SSGDAVFKFSPLDPER.R  369 - 388 2377.2330 2376.2257 2376.2802 -23 1 R.LFFQQVVSILQRSLPTTDER.H  421 - 440 2192.0940 2191.0867 2191.0871 -0 0 R.FMFIIQSLSALTGTYDEAGK.Q  467 - 476 1141.5990 1140.5917 1140.5564 31 0 K.AIGDYTFQAR.S  594 - 609 1641.6890 1640.6817 1640.7386 -35 1 R.SGSMADGEDKVAIAMK.A 2 Oxidation (M) |
| 39 | **Identification:** PMF  **GI number:** [gi|222085858](http://www.matrixscience.com/cgi/protein_view.pl?file=../data/20100721/FtemlGcTS.dat&hit=1)  **Product:** Translation elongation factor Ts [Agrobacterium radiobacter K84] **Gene:** *tsf*  **Mass:** 32268    **Score:** 77     **Expect:** 0.069  **Matches:** 8 Nominal mass (M_r_): **32268**; Calculated pI value: **5.15**  Number of mass values searched: **19** Number of mass values matched: **8** Sequence Coverage: **29%**  Start - End Observed Mr(expt) Mr(calc) ppm Miss Sequence  71 - 85 1648.9320 1647.9247 1647.8468 47 0 K.AVVVELNSETDFVAR.N  128 - 140 1404.7690 1403.7617 1403.7078 38 0 K.DAIATIGENMTLR.R  128 - 140 1420.7570 1419.7497 1419.7028 33 0 K.DAIATIGENMTLR.R Oxidation (M)  145 - 166 2250.2960 2249.2887 2249.1440 64 0 K.LEVEHGVVATYIHNAAGDGIGK.L  180 - 187 816.4690 815.4617 815.4865 -30 0 K.AVLTSIGR.Q  188 - 202 1605.9570 1604.9497 1604.8820 42 0 R.QVAMHIAATNPLAIR.A  203 - 213 1159.5850 1158.5777 1158.5517 23 0 R.AEEVDAAVAER.E  216 - 223 992.5190 991.5117 991.5087 3 0 R.NVFIEQSR.E |
| 40 | **Identification:** PMF  **GI number:** [gi|227821753](http://www.matrixscience.com/cgi/protein_view.pl?file=../data/20100729/FtepfnSem.dat&hit=1)  **Product:** Elongation factor G [*Rhizobium* sp. NGR234] **Gene:** *fusA*  **Mass:** 77966    **Score:** **92**  **Expect:** 0.002  **Matches:** 12  ] Nominal mass (M_r_): **77966**; Calculated pI value: **5.17**  Number of mass values searched: **25** Number of mass values matched: **12** Sequence Coverage: **22%**  Start - End Observed Mr(expt) Mr(calc) ppm Miss Sequence  82 - 99 2102.1330 2101.1257 2101.0480 37 0 R.FNIIDTPGHVDFTIEVER.S  259 - 269 1342.6320 1341.6247 1341.5999 19 0 K.FHPMFCGTAFK.N  321 - 334 1567.7840 1566.7767 1566.7865 -6 0 K.IMNDPFVGSLTFAR.I  321 - 334 1583.7820 1582.7747 1582.7814 -4 0 K.IMNDPFVGSLTFAR.I Oxidation (M)  387 - 405 2158.1730 2157.1657 2157.0987 31 0 K.ETTTGDTLCDPLKPVILER.M  435 - 443 1005.6120 1004.6047 1004.4927 112 0 R.LAAEDPSFR.V  444 - 469 2839.6300 2838.6227 2838.4433 63 1 R.VKTDEESGQTIIAGMGELHLDIIVDR.M  446 - 469 2612.1530 2611.1457 2611.2799 -51 0 K.TDEESGQTIIAGMGELHLDIIVDR.M  473 - 488 1750.9020 1749.8947 1749.9050 -6 1 R.EFKVEASVGAPQVAYR.E  569 - 591 2434.5560 2433.5487 2433.2176 136 0 K.ATLIDGAFHDVDSSVLAFEIASR.A  612 - 631 2176.2310 2175.2237 2175.0696 71 0 K.VEVVTPEDYVGDVIGDLNSR.R  612 - 632 2332.2660 2331.2587 2331.1707 38 1 K.VEVVTPEDYVGDVIGDLNSRR.G |
| 41 | **Identification:** PMF  **GI number:** [gi|86355771](http://www.matrixscience.com/cgi/protein_view.pl?file=../data/20100729/FtepfGcah.dat&hit=1)  **Product:** Polynucleotide phosphorylase/polyadenylase [Rhizobium etli CFN 42] **Gene:** *pnp*  **Mass:** 77491    **Score:** **86**  **Expect:** 0.0072  **Matches:** 9  Nominal mass (M_r_): **77491**; Calculated pI value: **5.20**  Number of mass values searched: **15** Number of mass values matched: **9** Sequence Coverage: **13%**  Start - End Observed Mr(expt) Mr(calc) ppm Miss Sequence  81 - 93 1473.6700 1472.6627 1472.7219 -40 0 R.EGRPSENETLVSR.L  338 - 346 989.4630 988.4557 988.5090 -54 0 R.THGSALFTR.G  522 - 531 956.4760 955.4687 955.5563 -92 1 K.VALGQAKGGR.A  547 - 555 974.4630 973.4557 973.4981 -44 0 R.GQLGEFAPR.I  588 - 597 1103.5140 1102.5067 1102.5506 -40 0 K.INIEDDGTVK.I  633 - 646 1519.7020 1518.6947 1518.7256 -20 0 K.TADFGAFVNFFGAR.D  647 - 659 1424.6980 1423.6907 1423.7419 -36 0 R.DGLVHISQLASER.V  663 - 672 1091.5150 1090.5077 1090.5506 -39 1 K.TTDVVKEGDK.V  677 - 683 849.4000 848.3927 848.4392 -55 0 K.LLGFDER.G |
| 42 | **Identification:** PMF  **GI number:** [gi|294624706](http://www.matrixscience.com/cgi/protein_view.pl?file=../data/20100817/FtepIbcnL.dat&hit=1)  **Product:** Translation initiation factor IF-2 [*Xanthomonas fuscans* subsp. *aurantifolii* str. ICPB 11122] **Gene:** *infB*  **Mass:** 83626    **Score:** **79**  **Expect:** 0.04  **Matches:** 7  Nominal mass (M_r_): **83626**; Calculated pI value: **5.89**  Number of mass values searched: **9** Number of mass values matched: **7** Sequence Coverage: **10%**  Start - End Observed Mr(expt) Mr(calc) ppm Miss Sequence  19 - 27 1200.6940 1199.6867 1199.6007 72 1 R.QRNLEEQQR.L  129 - 145 1786.8920 1785.8847 1785.8316 30 1 R.GSHVMVAGVEDDDATKR.F  206 - 225 2083.1130 2082.1057 2082.1936 -42 1 R.EVAIGETITVADLAQKLALK.G  491 - 503 1556.7090 1555.7017 1555.7565 -35 1 K.RGDYLVCGIQYGR.V  708 - 722 1552.8530 1551.8457 1551.7789 43 0 K.FGAVAGCMVIEGVVK.R Oxidation (M)  708 - 723 1692.8620 1691.8547 1691.8851 -18 1 K.FGAVAGCMVIEGVVKR.S  756 - 765 1034.6280 1033.6207 1033.4863 130 0 R.NGTECGIGVK.A |
| 43 | **Identification:** PMF  **GI number:** [gi|218672404](http://www.matrixscience.com/cgi/protein_view.pl?file=../data/20100720/FtemifSEL.dat&hit=1)  **Product:** Elongation factor EF-Tu protein [*Rhizobium etli* GR56]  **Mass:** 31884    **Score:** **130**  **Expect:** 3.2e-07  **Matches:** 12   Sequence Coverage: **50%**  Nominal mass (M_r_): **31884**; Calculated pI value: **4.87**  Start - End Observed Mr(expt) Mr(calc) ppm Miss Sequence  1 - 13 1421.6050 1420.5977 1420.5897 6 0 -.MCSAADGPMPQTR.E  14 - 20 851.5080 850.5007 850.5025 -2 0 R.EHILLAR.Q  21 - 33 1383.6720 1382.6647 1382.8286 -118 0 R.QVGVPAIVVFLNK.V  34 - 51 2084.1660 2083.1587 2083.0685 43 0 K.VDQVDDAELLELVELEVR.E  52 - 68 1893.9850 1892.9777 1892.9408 20 0 R.ELLSSYDFPGDDIPVVK.G  69 - 80 1176.4750 1175.4677 1175.5670 -84 0 K.GSALAALEDSDK.K  137 - 151 1612.7220 1611.7147 1611.8832 -105 0 K.VGEEVEIVGIRPTSK.T  152 - 161 1140.4690 1139.4617 1139.5645 -90 0 K.TTVTGVEMFR.K  162 - 178 1767.9480 1766.9407 1766.9639 -13 1 R.KLLDQGQAGDNIGALVR.G  163 - 178 1639.8790 1638.8717 1638.8689 2 0 K.LLDQGQAGDNIGALVR.G  203 - 212 1186.4950 1185.4877 1185.6104 -103 0 K.FMAEAYILTK.E  218 - 232 2021.0100 2020.0027 2019.9744 14 0 R.HTPFFTNYRPQFYFR.T |
| 44 | **Identification:** PMF  **GI number:** [gi|89056301](http://www.matrixscience.com/cgi/protein_view.pl?file=../data/20100726/FtemCGsem.dat&hit=1)  **Product:** LysR family transcriptional regulator [*Jannaschia* sp. CCS1]  **Mass:** 32077    **Score:** **86**  **Expect:** 0.0083  **Matches:** 6   Nominal mass (M_r_): **32077**; Calculated pI value: **5.57**  Number of mass values searched: **10** Number of mass values matched: **6** Sequence Coverage: **25%**  Start - End Observed Mr(expt) Mr(calc) ppm Miss Sequence  10 - 24 1742.0040 1740.9967 1740.8505 84 0 K.QLEAFVCVVDTGTFR.K  10 - 25 1870.1050 1869.0977 1868.9455 81 1 K.QLEAFVCVVDTGTFRK.A  26 - 40 1499.8530 1498.8457 1498.8103 24 0 K.AAGILGTTQPNISTR.I  41 - 61 2266.9980 2265.9907 2266.2103 -97 1 R.IAALEDVLGTILMHRDAGSIR.V Oxidation (M)  119 - 125 833.3720 832.3647 832.4443 -96 0 K.DLYPAVR.V  277 - 293 1728.9470 1727.9397 1727.9206 11 1 R.119.- |
| 45 | **Identification:** PMF  **GI number:** [gi|159184760](http://www.matrixscience.com/cgi/protein_view.pl?file=../data/20100817/FtepIxETR.dat&hit=1)  **Product:** AraC family transcriptional regulator [*Agrobacterium tumefaciens* str. C58]  **Mass:** 27498    **Score:** **78**  **Expect:** 0.0002  **Matches:** 5   Nominal mass (M_r_): **27498**; Calculated pI value: **7.11**  Number of mass values searched: **8** Number of mass values matched: **5** Sequence Coverage: **21%**  Start - End Observed Mr(expt) Mr(calc) ppm Miss Sequence  1 - 12 1383.8190 1382.8117 1382.6976 83 1 -.MTVASADIRTYR.Q  2 - 12 1252.7090 1251.7017 1251.6571 36 1 M.TVASADIRTYR.Q  20 - 34 1658.9150 1657.9077 1657.9417 -20 0 R.HGFVQIVLPVSGHLR.I  202 - 213 1298.8190 1297.8117 1297.7241 68 0 R.ESSLPIAEIALR.S  225 - 238 1488.9350 1487.9277 1487.7844 96 1 R.ALRNAVGETPAAYR.R |
| 46 | **Identification:** PMF  **GI number:** [gi|222081230](http://www.matrixscience.com/cgi/protein_view.pl?file=../data/20100817/FtepIzHtR.dat&hit=1)  **Product:** Transcriptional regulator protein [*Agrobacterium radiobacter* K84]  **Mass:** 98220    **Score:** **78**  **Expect:** 0.00024  **Matches:** 7   Nominal mass (M_r_): **98220**; Calculated pI value: **6.38**  Number of mass values searched: **10** Number of mass values matched: **7** Sequence Coverage: **10%**  Start - End Observed Mr(expt) Mr(calc) ppm Miss Sequence  34 - 46 1346.9000 1345.8927 1345.7718 90 1 R.RLTTIVAPAGYGK.T  97 - 115 1889.0140 1888.0067 1887.9724 18 0 K.GSDANSMPVASLLSALVTR.L  156 - 165 1171.8550 1170.8477 1170.6873 137 1 K.FVLISRSPPR.F  166 - 178 1476.0060 1474.9987 1474.8143 125 1 R.FPTSALRLNSELK.Q  625 - 644 1996.9640 1995.9567 1996.0159 -30 1 R.LNGEIMAAHAAIDSARSAAK.R  647 - 653 876.6140 875.6067 875.4977 124 1 R.GFDRLIR.L  859 - 868 1131.7630 1130.7557 1130.6295 112 1 R.KLDLSNNTVK.F |
| 47 | **Identification:** PMF  **GI number:** [gi|190895600](http://www.matrixscience.com/cgi/protein_view.pl?file=../data/20100729/FtepfGsEO.dat&hit=1)  **Product:** Probable transcriptional regulator protein, AraC family [*Rhizobium etli* CIAT 652]  **Mass:** 42937    **Score:** **84**  **Expect:** 0.013  **Matches:** 5   Nominal mass (M_r_): **42937**; Calculated pI value: **6.91** Number of mass values searched: **5** Number of mass values matched: **5** Sequence Coverage: **11%**  Number of mass values searched: **5** Number of mass values matched: **5** Sequence Coverage: **11%**  Start - End Observed Mr(expt) Mr(calc) ppm Miss Sequence  116 - 131 1779.9630 1778.9557 1778.9176 21 1 R.SSPIDHWNVGVLRSGR.T  132 - 138 877.3340 876.3267 876.4090 -94 0 R.TWTEANR.H  152 - 158 855.3690 854.3617 854.4286 -78 0 R.SLGYPYR.G  350 - 363 1565.9350 1564.9277 1564.8031 80 1 R.KTIATEHPAMPPTR.A Oxidation (M)  351 - 363 1421.8890 1420.8817 1420.7133 119 0 K.TIATEHPAMPPTR.A |
| 48 | **Identification:** PMF  **GI number:** [gi|222106418](http://www.matrixscience.com/cgi/protein_view.pl?file=../data/20100802/FtepriHTt.dat&hit=1)  **Product:** Transcriptional regulator GntR family [*Agrobacterium vitis* S4]  **Mass:** 26366    **Score:** **81**  **Expect:** 0.024  **Matches:** 5   Nominal mass (M_r_): **26366**; Calculated pI value: **5.82**  Number of mass values searched: **7** Number of mass values matched: **5** Sequence Coverage: **32%**  Start - End Observed Mr(expt) Mr(calc) ppm Miss Sequence  1 - 16 1868.6870 1867.6797 1867.9210 -129 1 -.MIDDHDNSNLALNKLR.E  112 - 126 1625.5870 1624.5797 1624.7767 -121 0 R.ANATDVQAMYDLVAK.I Oxidation (M)  147 - 167 2412.9340 2411.9267 2412.2042 -115 1 K.IAQCAGNSLFLALFDVMNRTR.Q Oxidation (M)  214 - 225 1421.6890 1420.6817 1420.8402 -112 0 R.VHLLTLQESLIR.I  226 - 238 1326.5630 1325.5557 1325.6827 -96 0 R.ITSIDTAVHVAEA.- |
| 49 | **Identification:** PMF  **GI number:** [gi|222106466](http://www.matrixscience.com/cgi/protein_view.pl?file=../data/20100816/FtepIaeeT.dat&hit=1)  **Product:** Transcriptional regulator ROK family [*Agrobacterium vitis* S4]  **Mass:** 41156    **Score:** **86**  **Expect:** 0.0075  **Matches:** 5] Nominal mass (M_r_): **41156**; Calculated pI value: **7.03**  Number of mass values matched: **5** Sequence Coverage: **18%**  Start - End Observed Mr(expt) Mr(calc) ppm Miss Sequence  143 - 160 1808.8760 1807.8687 1807.9866 -65 0 K.VLLAAVAMPGVIDPETGR.L  270 - 286 1791.8300 1790.8227 1790.8734 -28 1 R.GILERYGNPGDGGMTVR.D  275 - 294 2092.9600 2091.9527 2091.9895 -18 1 R.YGNPGDGGMTVRDILEAAEK.G  295 - 308 1430.7070 1429.6997 1429.7525 -37 1 K.GDAKALATVQETAR.L  299 - 325 2790.1680 2789.1607 2789.5473 -139 1 K.ALATVQETARLAALLVVSVHAMLDPGK.I Oxidation (M) |
| 50 | **Identification:** MS/MS  **GI number:**[gi|222082875](http://www.matrixscience.com/cgi/protein_view.pl?file=../data/20100817/FtepIzaet.dat&hit=gi|222082875&db_idx=1&px=1&ave_thresh=26&_ignoreionsscorebelow=0&report=0&_sigthreshold=0.05&_msresflags=1025&_msresflags2=2&percolate=-1&percolate_rt=0)  **Product:** Transcriptional regulator, MarR family [*Agrobacterium radiobacter* K84]  **Mass:** 18141    **Score:** 29     **Matches:** 1(1)  **Sequences:** 1(1)   Nominal mass (M_r_): **18141**; Calculated pI value: **5.46** Sequence Coverage: **4%**  Start - End Observed Mr(expt) Mr(calc) ppm Miss Sequence  123 - 129 800.5310 799.5237 799.5028 26 1 R.VRGIVTR.Q ([Ions score 29](http://www.matrixscience.com/cgi/peptide_view.pl?file=../data/20100817/FtepIzaet.dat&query=1&hit=1&index=gi|222082875&px=1&section=5&ave_thresh=26&_ignoreionsscorebelow=0&report=0&_sigthreshold=0.05&_msresflags=1025&_msresflags2=2&percolate=-1&percolate_rt=0)) |
| 51 | **Identification:** PMF  **GI number:** [gi|222084927](http://www.matrixscience.com/cgi/protein_view.pl?file=../data/20100817/FtepIbawe.dat&hit=1)  **Product:** ATP-dependent RNA helicase protein [*Agrobacterium radiobacter* K84]  **Mass:** 69955    **Score:** **82**  **Expect:** 8.7e-05  **Matches:** 6   Nominal mass (M_r_): **69955**; Calculated pI value: **9.17**  Number of mass values searched: **8** Number of mass values matched: **6** Sequence Coverage: **15%**  Start - End Observed Mr(expt) Mr(calc) ppm Miss Sequence  1 - 18 1945.0660 1944.0587 1944.0502 4 1 -.MTEFEGIVPAIAQALAKR.G  196 - 202 893.3190 892.3117 892.4402 -144 0 K.LAENYQR.D  223 - 240 1980.0600 1979.0527 1979.1163 -32 1 R.ALTVVPSDRENAIINVLR.Y  307 - 334 3019.7560 3018.7487 3018.6251 41 1 R.GIDLPGLDLVIHADIPTNPETLLHRSGR.T  341 - 353 1296.8650 1295.8577 1295.7925 50 1 R.KGVSALIVPLSGR.R  613 - 627 1442.9230 1441.9157 1441.7273 131 1 K.SDAGSPAGKPNSRAK.R |
| 52 | **Identification:** PMF  **GI number:** [gi|222086102](http://www.matrixscience.com/cgi/protein_view.pl?file=../data/20100816/FtepIfsSh.dat&hit=1)  **Product:** FeS assembly ATPase SufC [Agrobacterium radiobacter K84] **Gene:** *sufC*  **Mass:** 27375    **Score:** **77**  **Expect:** 0.00027  **Matches:** 4   Nominal mass (M_r_): **27375**; Calculated pI value: **5.08**  Sequence Coverage: **18%**  Start - End Observed Mr(expt) Mr(calc) ppm Miss Sequence  43 - 52 1080.7210 1079.7137 1079.5975 108 0 K.STLSYILAGR.D  142 - 153 1260.7670 1259.7597 1259.6622 77 0 K.RPLNVGFSGGEK.K  199 - 208 1199.7930 1198.7857 1198.6822 86 0 R.AVIVITHYQR.L  209 - 223 1788.0300 1787.0227 1786.9869 20 0 R.LLDYIVPDTVHVLYK.G |
| 53 | **Identification:** PMF  **GI number:** [gi|222082138](http://www.matrixscience.com/cgi/protein_view.pl?file=../data/20100723/FtemIrawe.dat&hit=1)  **Product:** Chloride peroxidase protein [*Agrobacterium radiobacter* K84]  **Mass:** 34965    **Score:** **82**  **Expect:** 0.019  **Matches:** 8   Nominal mass (M_r_): **34965**; Calculated pI value: **7.88**  Number of mass values searched: **24** Number of mass values matched: **8** Sequence Coverage: **23%**  Start - End Observed Mr(expt) Mr(calc) ppm Miss Sequence  138 - 153 1504.7830 1503.7757 1503.7543 14 0 K.GAIHVGHSTGGGEVAR.Y  154 - 163 1107.5980 1106.5907 1106.5257 59 0 R.YVAQYGGHGR.V  180 - 196 1861.9640 1860.9567 1860.9370 11 1 K.SEKNPGGLPLEVFDGFR.S  183 - 196 1517.7990 1516.7917 1516.7674 16 0 K.NPGGLPLEVFDGFR.S  209 - 220 1353.7050 1352.6977 1352.6513 34 0 R.DIPAGPFYGFNR.S  244 - 250 906.4920 905.4847 905.4065 86 0 K.AHYDCIK.A  251 - 262 1402.6910 1401.6837 1401.6300 38 0 K.AFSETDFTEDLK.K  251 - 263 1530.7970 1529.7897 1529.7249 42 1 K.AFSETDFTEDLKK.I |
| 54 | **Identification:** MS/MS  **GI number:** [gi|186472508](http://www.matrixscience.com/cgi/protein_view.pl?file=../data/20100817/FtepIxHOS.dat&hit=gi|186472508&db_idx=1&px=1&ave_thresh=46&_ignoreionsscorebelow=0&report=0&_sigthreshold=0.05&_msresflags=1025&_msresflags2=2&percolate=-1&percolate_rt=0)  **Product:** Flavoprotein WrbA [*Burkholderia phymatum* STM815]  **Mass:** 20930    **Score:** 50     **Matches:** 1(1)  **Sequences:** 1(1)   Nominal mass (M_r_): **20930**; Calculated pI value: **6.19**  Sequence Coverage: **13%**  Start - End Observed Mr(expt) Mr(calc) ppm Miss Sequence  54 - 79 2744.3980 2743.3907 2743.4028 -4 0 K.LDQQAPIATVADLEQYDAIVVGTGTR.Y ([Ions score 50](http://www.matrixscience.com/cgi/peptide_view.pl?file=../data/20100817/FtepIxHOS.dat&query=1&hit=1&index=gi|186472508&px=1&section=5&ave_thresh=46&_ignoreionsscorebelow=0&report=0&_sigthreshold=0.05&_msresflags=1025&_msresflags2=2&percolate=-1&percolate_rt=0)) |
| 55 | **Identification:** PMF  **GI number:** [gi|170699364](http://www.matrixscience.com/cgi/protein_view.pl?file=../data/20100727/FtemCbcTO.dat&hit=1)  **Product:** NADPH-dependent FMN reductase [*Burkholderia ambifaria* IOP40-10]  **Mass:** 8539     **Score:** **84**  **Expect:** 0.011  **Matches:** 4   Nominal mass (M_r_): **8539**; Calculated pI value: **6.71**  Number of mass values searched: **5** Number of mass values matched: **4** Sequence Coverage: **78%**  Start - End Observed Mr(expt) Mr(calc) ppm Miss Sequence  2 - 19 1779.1270 1778.1197 1777.9435 99 0 M.LGTSPGATGTALAQQHLR.N  29 - 43 1770.0140 1769.0067 1768.8930 64 1 K.TLAQPEMFIKHDPAR.I Oxidation (M)  39 - 56 2052.2090 2051.2017 2050.9668 115 1 K.HDPARIDDQGQIVSEDTR.K  58 - 71 1800.1650 1799.1577 1798.9155 135 1 K.FLQGFVDRYVDWVR.M |
| 56 | **Identification:** PMF  **GI number:** [gi|194431754](http://www.matrixscience.com/cgi/protein_view.pl?file=../data/20100729/FtemOeYmt.dat&hit=1)  **Product:** 2,5-diketo-d-gluconic acid reductase a [Shigella dysenteriae 1012]  **Mass:** 19399    **Score:** **80**  **Expect:** 0.031  **Matches:** 5  Nominal mass (M_r_): **19399**; Calculated pI value: **6.22**  Number of mass values searched: **17** Number of mass values matched: **5** Sequence Coverage: **24%**  Start - End Observed Mr(expt) Mr(calc) ppm Miss Sequence  17 - 28 1358.7160 1357.7087 1357.7639 -41 1 K.GMIELQKEGLIK.S  99 - 106 909.4500 908.4427 908.4603 -19 1 R.DLADKYGK.T  144 - 154 1230.6070 1229.5997 1229.6503 -41 1 R.LDKDELGEIAK.L  147 - 154 874.4330 873.4257 873.4443 -21 0 K.DELGEIAK.L  161 - 170 1002.5920 1001.5847 1001.4455 139 0 R.LGPDPDQFGG.- |
| 57 | **Identification:** PMF  **GI number:** [gi|222085370](http://www.matrixscience.com/cgi/protein_view.pl?file=../data/20100720/FtemixeES.dat&hit=1)  **Product:** Ferredoxin reductase protein [*Agrobacterium radiobacter* K84]  **Mass:** 43777    **Score:** **80**  **Expect:** 0.035  **Matches:** 8   Nominal mass (M_r_): **43777**; Calculated pI value: **5.88**  Sequence Coverage: **21%**  Start - End Observed Mr(expt) Mr(calc) ppm Miss Sequence  5 - 20 1485.7850 1484.7777 1484.8715 -63 0 R.LVIVGAGQAGFALAAK.V  5 - 22 1740.9530 1739.9457 1740.0410 -55 1 R.LVIVGAGQAGFALAAKVR.A  49 - 59 1358.6720 1357.6647 1357.6700 -4 1 K.KYLLGEMAFDR.L Oxidation (M)  50 - 59 1230.5750 1229.5677 1229.5750 -6 0 K.YLLGEMAFDR.L Oxidation (M)  60 - 76 2172.0150 2171.0077 2171.0912 -38 0 R.LLFRPEHWYADNNVEIR.L  148 - 165 1743.0040 1741.9967 1742.0090 -7 0 R.VLIIGGGYIGLEAAAVAR.H  168 - 179 1362.6770 1361.6697 1361.6861 -12 0 R.GLEVTLIEMADR.I Oxidation (M)  350 - 359 1173.5730 1172.5657 1172.5502 13 0 R.EGSASVWYFK.E |
| 58 | **Identification:** PMF  **GI number:** [gi|222149801](http://www.matrixscience.com/cgi/protein_view.pl?file=../data/20100720/Ftemixstt.dat&hit=1)  **Product:** Hypothetical protein Avi_3814 [*Agrobacterium vitis* S4]  **Mass:** 24632    **Score:** **78**  **Expect:** 0.05  **Matches:** 5   Nominal mass (M_r_): **24632**; Calculated pI value: **5.03**  Sequence Coverage: **32%**  Start - End Observed Mr(expt) Mr(calc) ppm Miss Sequence  41 - 52 1158.7660 1157.7587 1157.7020 49 0 K.TGALVSVLLGTK.T  53 - 63 1030.6880 1029.6807 1029.5931 85 1 K.TGRSLAGGALK.V  122 - 138 1770.8950 1769.8877 1769.8366 29 1 K.AMIAAAKADGHIDEGER.A Oxidation (M)  160 - 176 1754.8830 1753.8757 1753.8846 -5 0 R.AELDNPTDLDALVAAAR.T  210 - 228 1878.0900 1877.0827 1877.0258 30 0 R.LGLADGLIDHIEATVAAAK.V |
| 59 | **Identification:** PMF  **GI number:** [gi|209547526](http://www.matrixscience.com/cgi/protein_view.pl?file=../data/20100817/FtepIzEtt.dat&hit=1)  **Product:** Hypothetical protein Rleg2_5527 [*Rhizobium leguminosarum* bv. *trifolii* WSM2304]  **Mass:** 33584    **Score:** **80**  **Expect:** 0.035  **Matches:** 6   Nominal mass (M_r_): **33584**; Calculated pI value: **6.02**  Number of mass values searched: **13**  Number of mass values matched: **6**  Sequence Coverage: **28%**  Start - End Observed Mr(expt) Mr(calc) ppm Miss Sequence  1 - 17 1852.1420 1851.1347 1850.9383 106 0 -.MATVFQGPSVLSMSKPR.A Oxidation (M)  34 - 57 2634.6540 2633.6467 2633.5421 40 1 R.VLRPLVKLALASGFNFTAFTTVLR.R  59 - 73 1764.0180 1763.0107 1762.9505 34 1 R.LYIEVAEKEFALPNK.Q  225 - 238 1474.8640 1473.8567 1473.7034 104 0 R.AVHYDGMSPALAAR.L Oxidation (M)  272 - 283 1467.9440 1466.9367 1466.7228 146 0 R.WIAGLYVMTEER.E  272 - 285 1752.9450 1751.9377 1751.8665 41 1 R.WIAGLYVMTEERER.D |
